# The association between interleukin-6 gene -174G/C single nucleotide polymorphism and sepsis: an updated meta-analysis with trial sequential analysis

**DOI:** 10.1186/s12881-019-0766-2

**Published:** 2019-02-19

**Authors:** Yao Chen, Yanyan Hu, Zhenju Song

**Affiliations:** Department of Emergency Medicine, Zhongshan Hospital, Fudan University, 180 Fenglin Road, Shanghai, 200032 China

**Keywords:** Gene, Polymorphism, Interleukin-6, Sepsis, Meta-analysis, Trial sequential analysis

## Abstract

**Background:**

This article intends to explore the association between interleukin-6 gene (*IL-6*) -174 G/C single nucleotide polymorphism (SNP) and the risk and mortality of sepsis by conducting this updated meta-analysis with trial sequential analysis.

**Methods:**

References were made to PubMed, Web of Science, China National Knowledge Infrastructure for studies available by September 2018. Each publication was screened for its eligibility and data accessible. Statistical analysis was conducted on Stata 14.1 and TSA software 0.9.5.10 Beta

**Results:**

Twenty studies (including 3282 cases and 4926 controls) and eight studies (including 610 cases and 1856 controls) were respectively enrolled in the analysis on the association between *IL-6*-174 G/C polymorphism and the risk and mortality of sepsis. The results did not present any association between *IL-6*-174 G/C polymorphism and the risk and mortality of sepsis. An exception was that *IL-6*-174 G/C polymorphism was correlated with worse outcome in non-adults in recessive model, co-dominant model (CC vs. GG) and allelic model, while trial sequential analysis revealed it could be a false positive result nevertheless.

**Conclusions:**

*IL-6*-174 G/C polymorphism is not associated with the risk and mortality of sepsis. Trial sequential analysis showed that a large sample size was needed to get a more reliable result of the association between *IL-6*-174 G/C polymorphism and sepsis in non-adults.

**Electronic supplementary material:**

The online version of this article (10.1186/s12881-019-0766-2) contains supplementary material, which is available to authorized users.

## Background

Sepsis is a systemic multiorgan dysfunction secondary to the dysregulated host response to infection as suggested in the latest definition for sepsis (Sepsis-3.0) and has a strong correlation with intensive care unit (ICU) admission and in-hospital mortality [1, 2]. The recognition and management of sepsis has become a major issue in critical care medicine.

The alteration of immune function has been considered a key factor in the pathogenesis of sepsis [3]. The host immune system generates a series of substances such as cytokines in response to an infection or injury. IL-6 is a pro-inflammatory cytokine which is involved in the inflammatory reaction at the early stage of sepsis. It serves as a biomarker of the sepsis, and elevated serum IL-6 level indicates the deterioration of the disease and higher tendency to death [4, 5]. IL-6 blockade therapy is beneficial to the prognosis of sepsis by blocking systemic inflammatory response [6].

*IL*-6 gene, located at 7p15.3 is responsible for the regulation of the transcriptional activity during inflammation reaction. The possible association between its − 174 G/C polymorphism (rs1800795) at promoter region and the risk and mortality of sepsis has been widely studied. However, the findings are varied among different studies. *IL-6*-174 C allele is thought to block the norepinephrine-induced transcription factor binding to *IL-6* gene promotor and thus to lower the gene expression, which is favorable for inflammation-related diseases [7]. A meta-analysis by Chauhan M et al. in 2008 did not support the association between − 174 G/C polymorphism and the risk of sepsis in very low birth weight (VLBW) infants [8]. After that, a meta-analysis in 2013 demonstrated that *IL-6*-174 G/C polymorphism did not have a link with the risk and mortality of sepsis at any age and ethnicity groups [9]. In recent years, more studies on this topic have been published. A study found that *IL-6*-174 G allele was associated with early-onset sepsis in Saudi infants [10]. However, Mao Z et al. and Feng B et al. thought it was *IL-6*-174 C allele rather than G allele that contributed to the risk of sepsis induced by pneumonia [11, 12]. When it came to the mortality of septic patients, Lorente L et al. discovered better survival of septic patients with CC genotype [13]. Jimenez-Sousa MA et al. found the possible association between *IL-6*-174 CC genotype and a higher septic shock-related mortality in patients who underwent major surgery [14]. These studies have controversial findings, which made us curious how they would influence the results if they were included in a new meta-analysis. Thus, an updated meta-analysis was made, which was intended to add valuable information to the future studies.

## Methods

### Searching strategy

We searched PubMed, Web of Science and China National Knowledge Infrastructure (CNKI) for available studies published before September 2018. The keywords for searching were a combination of “IL-6, interleukin-6, rs1800795, -174G/C, polymorphism, sepsis, septicemia, septic shock”. Meanwhile, references of the relevant literature reviews were screened to identify potentially relevant publications.

### Criteria for inclusion and exclusion

**Fig. 1:**
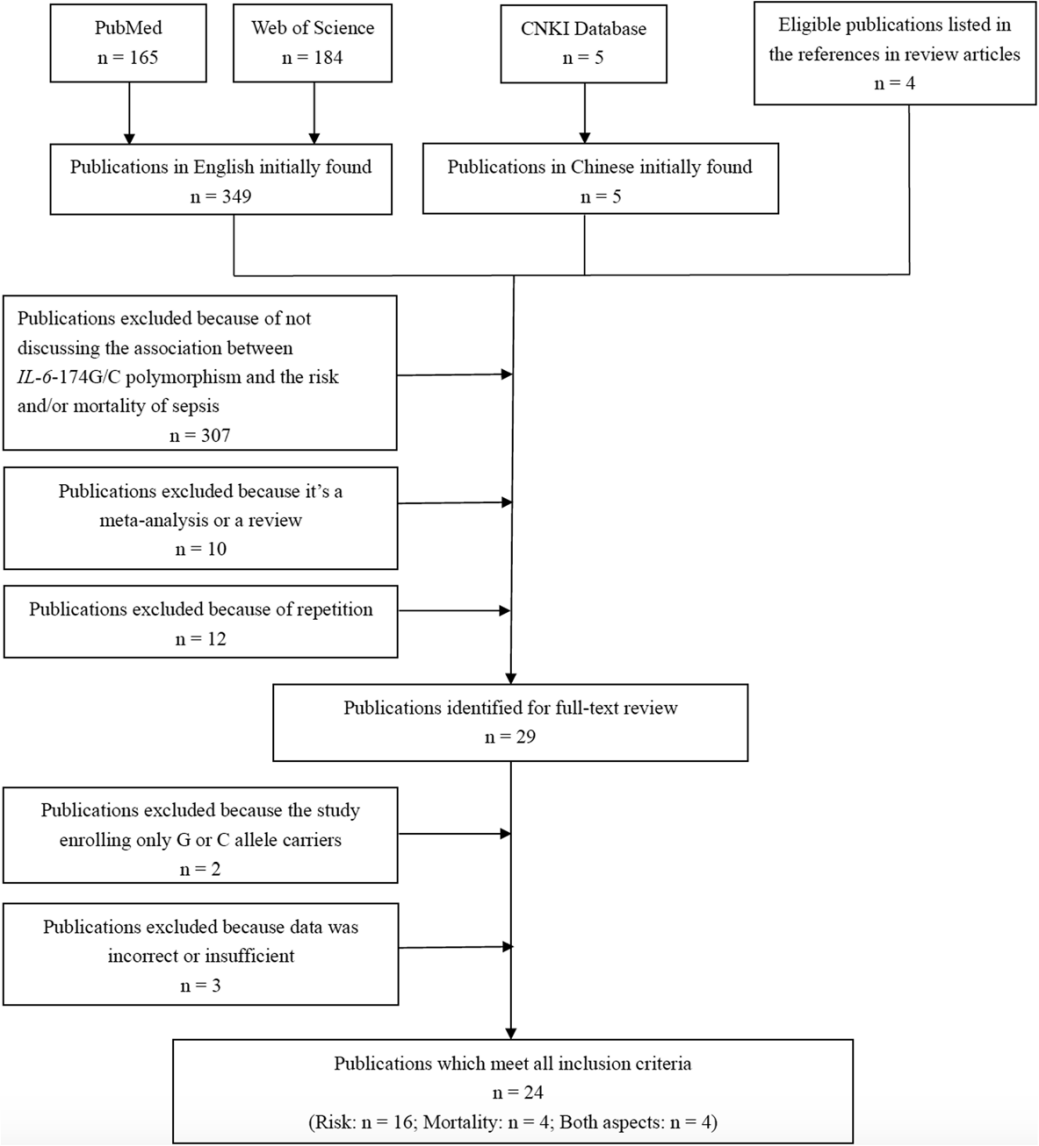
Flow diagram of the study selection process

The publications fulfilling the following criteria were included: an original study evaluating the association between *IL-6*-174 G/C polymorphism and the risk and/or mortality of sepsis; objects in each study were from same epoch; including a case group of sepsis; including a control group; including precise sample size of *IL-6*-174 G/C polymorphism of case and control groups which could be directly extracted or calculated according to the information available. Excluded were: repetition of the published studies; a meta-analysis or a review; study with insufficient or incorrect data; study with only G or C allele carriers. The screening process is illustrated in a flow chart (Fig. 1). All publications were identified by two reviewers independently. If two reviewers had opposite views, a third reviewer would be consulted for a decision.

### Quality assessment and data extraction

The quality of included studies was evaluated according to Newcastle-Ottawa Scale (NOS) by two researchers [15]. Available data were extracted by two authors independently. Disagreements were dealt with by a third reviewer. The following information was extracted from studies: first author, year of publication, country of the study and features of case and control groups such as ethnicity, age group, type of case and controls. Some studies discussed both the risk and mortality of sepsis. In this case, the information was collected respectively.

### Statistical analysis

The role of minor allele C in the risk and mortality of sepsis was targeted. Thus an analysis was made using dominant model (GC + CC versus GG), recessive model (CC versus GC + GG), co-dominant model (GC versus GG and CC versus GG) and allelic model (C versus G). While some studies only yielded a sum of number of genotypes, they were enrolled in analyses just under certain genetic models [16–19]. Statistical analysis was conducted on Stata 14.1. Each control group underwent goodness-of-fit χ^2^-test for evaluating Hardy-Weinberg equilibrium (HWE). When *P* > 0.05, the objects were under Hardy-Weinberg equilibrium and within a same Mendelian population. Homogeneity of the studies was tested. When *P* < 0.1 or *I*^*2*^ > 50%, a high level of heterogeneity between studies was envisaged and random-effect model was adopted; when *P* > 0.1 and *I*^*2*^ < 50%, there was no significant heterogeneity between studies, and fixed-effect model was used. The effect of *IL-6*-174 G/C on the risk or mortality of sepsis was measured by *P* value, odd ratio (OR) and 95% confidence interval (CI). *P* < 0.05 was reviewed as statistical significance. In the analysis on sepsis risk, subgroup analyses based on age group, ethnicity, restricted healthy controls were conducted. Subgroup analysis on age and ethnicity in the mortality analysis was also made. If no information of age group was given in a study, we considered it would be as a study on adult. The majority of the nation of study was taken as the ethnicity of the study population in case that it was not specifically mentioned. The objects in three studies were mainly Caucasians, so they were included in the subgroup analysis of the Caucasians as the number of non-Caucasians were very small [16, 20, 21]. Meanwhile, sensitivity analysis was conducted by repeating analysis after omitting one study each time to estimate the effect of quality of studies on the final result. Publication bias was evaluated by Egger’s test. Once *P* > 0.05 in regression test, no obvious publication bias would exist.

### Trial sequential analysis

Meta-analysis might be affected by type I error due to the increased risk of random error and repeated significance testing. Trial sequential analysis (TSA) was a useful tool to verify the reliability of the results from meta-analysis by estimating the required information size (RIS) (sample size of included studies) and calculating the threshold for statistical significance [22]. A type I error of 5%, power of 80%, relative risk reduction of 20% were defined and control event proportion was an average of each included study. If the Z-curve crossed RIS line, the result of meta-analysis would be conclusive. If the Z-curve crossed the O’Brien-Fleming boundary or futility boundary, the conclusions could be made even before it crossed the RIS line that *IL-6*-174G/C polymorphism have or did not have a correlation with sepsis, respectively. TSA was conducted in the genetic model with the most included studies. If each genetic model had the same number of included studies, TSA would be conducted in allelic model. Meta-analysis which presented a significant result in the pool analysis was also tested under TSA. TSA was conducted on TSA software 0.9.5.10 Beta.

## Results

### Searching results

354 studies were initially acquired from databases and 4 studies were from references of publications. After exclusion of irrelevant studies, review and meta-analysis as well as repetitions, 29 studies were identified for full-text review. 5 studies were further excluded after full-text review because of inadequate data for analysis [23–27]. Eventually, 24 studies fulfilled all criteria for inclusion, including 16 studies on the risk of sepsis, 4 studies on the mortality and 4 studies on both topics [10–14, 16–21, 28–40]. All included studies had a NOS score ≥ 6 (see Additional file 1: Table S1). Totally, there were 3282 cases and 4926 controls for the analysis on sepsis risk together with 610 cases and 1856 controls for the analysis on mortality. Main characteristics of all studies included as well as the genotype distributions of *IL-6*-174 G/C polymorphism were shown in Table 1.

**Table 1:** Main characteristics of the included studies

First author	Year	Country	Age group	Type of case	Type of control	Ethnicity	Case			Control			HWE^a^ in control
							GG	GC	CC	GG	GC	CC	
Schluter B	2002	USA	Adult	Sepsis, severe sepsis	Healthy	Caucasian	25	46	24	71	94	42	Yes
				Non-survivor	Survivor		2	17	6	11	8	6	Yes
Harding D	2003	UK	Infant	Septicemia	Preterm infant	Mainly Caucasian	24	27^b^		30	76^b^		N/A
Balding J	2003	Ireland	Adult	Meningococcal sepsis	Healthy	Caucasian	59	97	27	123	209	68	Yes
				Non-survivor	Survivor		13	10	2	46	87	25	Yes
Treszl A	2003	Hungary	Neonate	Sepsis	VLBW^c^ neonate	Caucasian	18	13	2	34	29	7	Yes
Barber RC	2004	USA	Adult	Severe sepsis	ICU patients with no or mild sepsis	Mixed	17	19^b^		69	54^b^		N/A
Ahrens P	2004	Germany	Infant	Sepsis	VLBW^c^ infants	Caucasian	24	21	5	97	177	32	No
Michalek J	2006	Czech Republic	aged 0–19 years	SIRS^d^, sepsis, severe sepsis, septic shock and MODS^e^	Healthy	Caucasian	103	172	48	160	345	139	Yes
McDaniel DO	2006	USA	not clear^f^	Sepsis	ICU patients	African American	16^g^		0	6^g^		0	N/A
						Caucasian	12^g^		3	13^g^		8	N/A
Sipahi T	2006	Turkey	aged 0–15 years	Severe sepsis	Healthy	not clear	26	14	4	52	19	6	No
				Non-survivor	Survivor		9	5	1	17	9	3	Yes
Baier RJ	2006	USA	Infant	Sepsis	VLBW^c^ infant	African American	94	18	2	110	9	0	Yes
						Caucasian	12	16	3	7	16	3	Yes
				Non-survivor	Survivor	African American	10	1	0	84	17	2	Yes
						Caucasian	1	1	1	11	15	2	Yes
Göpel W	2006	Germany	Infant	Sepsis	VLBW^c^ infant	Mainly Caucasian	29	50	18	128	143	49	Yes
Sabeinikovs O	2008	Latvia	Infant	Non-survivor	Survivor	Caucasian	10	21	13	20	32	7	Yes
Abdel-Hady H	2009	Egypt	Neonate	Sepsis	Neonates investigated for neonatal hyperbilirubinemia	Caucasian	17	26	11	28	32	11	Yes
				Non-survivor	Survivor		2	5	6	15	21	5	Yes
Solé-Violán J	2010	Spain	Adult	Severe sepsis and septic shock	CAP^h^	Caucasian	141	144	36	392	341	84	Yes
Davis SM	2010	USA	Female adult	Puerperal group streptococcus sepsis	Healthy	Caucasian	10	11	2	21	22	9	Yes
Carregaro F	2011	Brazil	Adult	Sepsis, severe sepsis and septic shock	Healthy	Mainly Caucasian^i^	49	39	9	94	96	17	Yes
Accardo Palumbo A	2011	Italy	aged 13–82 years	Sepsis	Burned patient	not clear	14^g^		2	22^g^		4	N/A
Watanabe E	2012	USA	Adult	Non-survivor	Survivor	Caucasian	113	149	46	245	363	125	Yes
Martín-Loeches I	2012	Spain	Adult	Sepsis, severe sepsis and septic shock	Healthy	Caucasian	581	516	130	438	413	102	Yes
Feng B	2015	China	Adult	Non-survivor	Survivor	Asian	42	20	1	139	72	3	Yes
Allam G	2015	Saudi Arabia	Infant	Early-onset sepsis	Healthy	not clear	39	20	10	13	32	23	Yes
Lorente L	2016	Spain	Adult	Non-survivor	Survivor	not clear	47	36	3	76	74	27	Yes
Mao ZR	2016	China	Adult	Sepsis	Healthy	Asian	56	37	95	97	66	37	No
Jimenez-Sousa MA	2017	Spain	Adult	Septic shock	Healthy	Not clear	93	90	19	43	51	13	Yes

^a^Hardy-Weinberg equilibrium

^b^GC and CC

^c^very low birth weight

^d^systemic inflammatory response syndrome

^e^multiple organ disorder syndrome

^f^patients in surgery intensive care unit

^g^GG and GC

^h^community-acquired pneumonia

^i^Caucasian ancestry mainly, together with small amount of Asian and African ancestry

### The association between *IL-6*-174 G/C polymorphism and the risk of sepsis

**Fig. 2:**
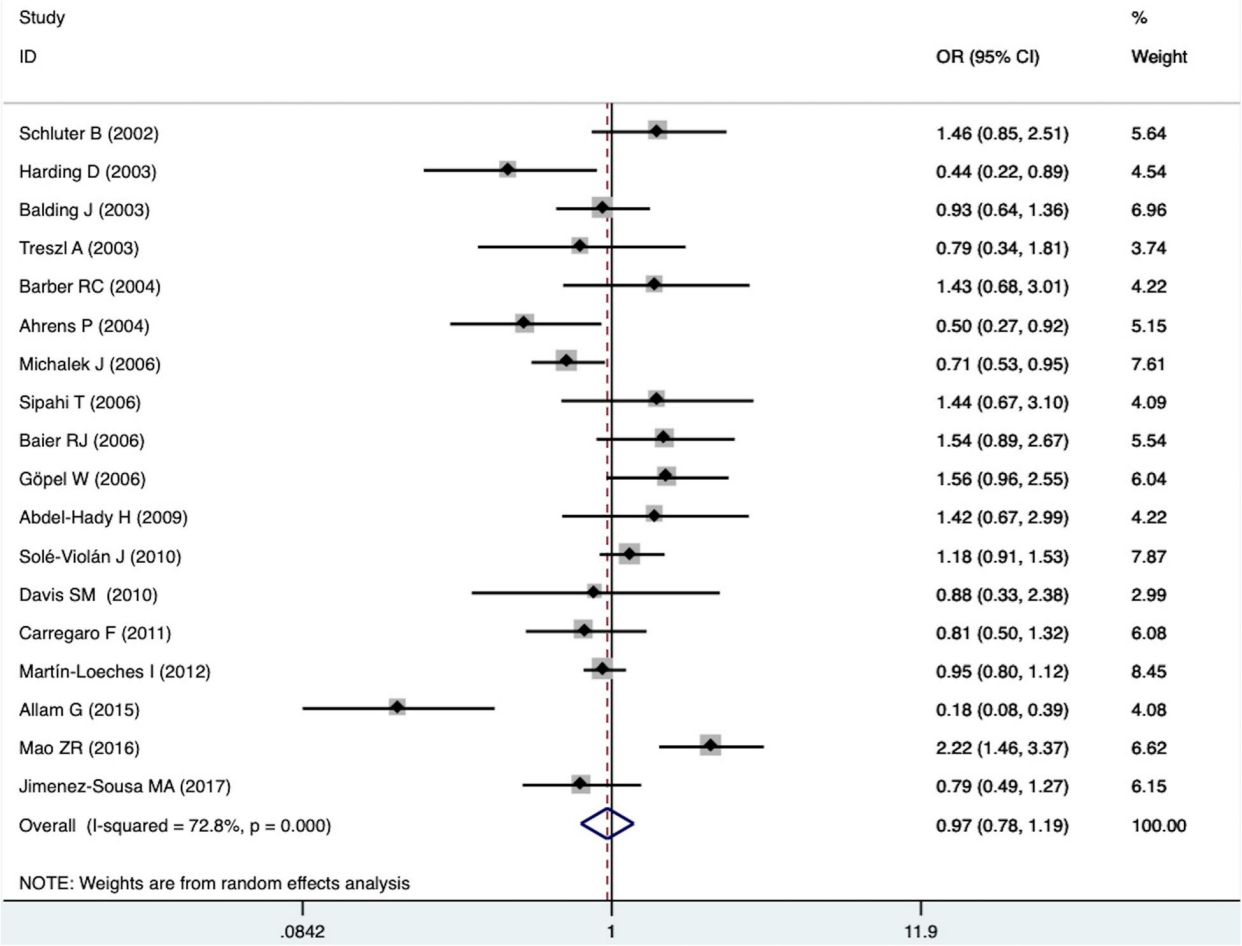
Forest plot of the association between *IL-6*-174 G/C polymorphism and the risk of sepsis under dominant model. The horizontal line represents the 95% confidence interval. And its length shows the range of the confidence interval and the size of the square in the middle shows the weight of the study. The diamond (and broken line) represents the overall summary estimate, with confidence interval given by its width. The unbroken vertical line is at the null value (OR = 1.0). CI, confidence interval

The results were shown in Table 2. The analysis resulted in no association between *IL-6*-174 G/C polymorphism and the risk of sepsis concerning the overall population under dominant model, recessive model, codominant model and allelic model (dominant model: *P* = 0.743, OR = 0.965, 95% CI: 0.782–1.192; recessive model: *P* = 0.96, OR = 0.992, 95% CI: 0.726–1.356; codominant model GC vs. GG: *P* = 0.57, OR = 0.950, 95% CI: 0.686–1.435; codominant model CC vs. GG: *P* = 0.966, OR = 0.992, 95% CI: 0.686–1.435; allelic model: *P* = 0.894, OR = 1.014, 95% CI: 0.831–1.236) (Fig. 2). When limiting control group to healthy ones or Mendelian population, the results remained unchanged. In the subgroup analysis on non-adults, adults and Caucasians, no association between *IL-6*-174 G/C polymorphism and the risk of sepsis was found, neither.

**Table 2:** Results of the meta-analysis, heterogeneity test and Egger’s test

	Meta-analysis			Heterogeneity test		Egger’s test
	no. of studies	OR (95% CI)	*P* value	*I* ^*2*^	*P* value	*P* value
The association between *IL-6* -174G/C polymorphism and the risk of sepsis						
Overall						
GC + CC vs GG	18	0.965 (0.782–1.192)	0.743	72.80%	< 0.001	0.694
CC vs. GC + GG	18	0.992 (0.726–1.356)	0.96	72.90%	< 0.001	0.81
GC vs. GG	16	0.950 (0.798–1.133)	0.57	52.00%	0.008	0.696
CC vs.GG	16	0.992 (0.686–1.435)	0.966	77.80%	< 0.001	0.989
C vs. G	16	1.014 (0.831–1.236)	0.894	84.20%	< 0.001	0.705
Non-adult						
GC + CC vs GG	9	0.796 (0.522–1.214)	0.29	78.40%	< 0.001	0.754
CC vs. GC + GG	8	0.785 (0.614–1.003)	0.053	41.50%	0.101	0.337
GC vs. GG	8	0.879 (0.578–1.336)	0.545	72.90%	0.001	0.694
CC vs.GG	8	0.779 (0.427–1.421)	0.415	69.60%	0.002	0.38
C vs. G	8	0.896 (0.643–1.248)	0.516	80.10%	< 0.001	0.475
Adult						
GC + CC vs GG	9	1.112 (0.900–1.374)	0.324	59.20%	0.012	0.464
CC vs. GC + GG	10	1.080 (0.700–1.666)	0.73	79.50%	< 0.001	0.73
GC vs. GG	9	0.988 (0.874–1.117)	0.851	0%	0.711	0.816
CC vs.GG	9	1.208 (0.766–1.905)	0.416	79.60%	< 0.001	0.904
C vs. G	9	1.124 (0.868–1.455)	0.375	86.40%	< 0.001	0.63
Caucasian						
GC + CC vs GG	16	0.857 (0.699–1.050)	0.136	65.20%	< 0.001	0.725
CC vs. GC + GG	17	0.892 (0.771–1.033)	0.127	6.70%	0.376	0.712
GC vs. GG	15	0.912 (0.756–1.099)	0.333	52.50%	0.009	0.954
CC vs.GG	15	0.853 (0.643–1.132)	0.271	55.00%	0.005	0.962
C vs. G	15	0.913 (0.787–1.059)	0.228	66.20%	< 0.001	0.821
Healthy control						
GC + CC vs GG	10	0.919 (0.686–1.232)	0.574	78.60%	< 0.001	0.709
CC vs. GC + GG	10	0.985 (0.623–1.557)	0.949	84.30%	< 0.001	0.979
GC vs. GG	10	0.894 (0.791–1.010)	0.072	47.30%	0.047	0.871
CC vs.GG	10	0.896 (0.531–1.511)	0.681	85.50%	< 0.001	0.995
C vs. G	10	0.964 (0.726–1.282)	0.803	89.30%	< 0.001	0.747
Mendelian population						
GC + CC vs GG	13	0,947 (0.768–1.167)	0.607	66.60%	< 0.001	0.701
CC vs. GC + GG	13	0.903 (0.777–1.049)	0.101	27.7%%	0.165	0.807
GC vs. GG	13	0.971 (0.805–1.171)	0.758	53.10%	0.012	0.645
CC vs.GG	13	0.880 (0.647–1.197)	0.416	61.50%	0.002	0.698
C vs. G	13	0.945 (0.806–1.109)	0.489	71.00%	< 0.001	0.81
The association between *IL-6* -174G/C polymorphism and the mortality of sepsis						
Overall						
GC + CC vs GG	10	1.050 (0.705–1.563)	0.812	57.90%	0.011	0.059
CC vs. GC + GG	10	1.299 (0.665–2.538)	0.444	62.20%	0.005	0.173
GC vs. GG	10	1.045 (0.709–1.540)	0.825	51.10%	0.031	0.143
CC vs.GG	10	1.340 (0.611–2.939)	0.465	67.00%	0.001	0.113
C vs. G	10	1.103 (0.795–1.531)	0.558	69.50%	0.001	0.134
Non-adult						
GC + CC vs GG	4	1.434 (0.810–2.538)	0.216	0%	0.449	0.883
CC vs. GC + GG	4	2.957 (1.442–6.066)	0.003	0%	0.434	0.684
GC vs. GG	4	1.098 (0.588–2.050)	0.77	0%	0.746	0.871
CC vs.GG	4	3.221 (1.431–7.250)	0.005	3%	0.38	0.872
C vs. G	4	1.631 (1.101–2.416)	0.015	31.90%	0.221	0.783
Adult						
GC + CC vs GG	6	1.005 (0.593–1.705)	0.985	73.10%	0.002	0.126
CC vs. GC + GG	6	1.204 (0.342–4.236)	0.772	86.50%	< 0.001	0.486
GC vs. GG	6	1.078 (0.639–1.818)	0.778	70.30%	0.005	0.127
CC vs.GG	6	0.868 (0.341–2.208)	0.766	66.60%	0.01	0.302
C vs. G	6	0.940 (0.654–1.352)	0.739	70.90%	0.004	0.377
Caucasian						
GC + CC vs GG	9	1.206 (0.723–2.007)	0.474	65.70%	0.003	0.094
CC vs. GC + GG	9	1.376 (0.656–2.884)	0.399	67.90%	0.002	0.097
GC vs. GG	9	1.143 (0.713–1.834)	0.579	55.50%	0.021	0.174
CC vs.GG	9	1.430 (0.598–3.420)	0.422	71.30%	< 0.001	0.071
C vs. G	9	1.180 (0.802–1.737)	0.401	73.50%	< 0.001	0.101

### The association between *IL-6*-174 G/C polymorphism and the mortality of sepsis

The results were shown in Table 2. In the analysis of overall population, *IL-6*-174 G/C polymorphism did not appear to be associated with the mortality of sepsis in dominant model (*P* = 0.812, OR = 1.050, 95% CI: 0.705–1.563), recessive model (*P* = 0.444, OR = 1.299, 95% CI: 0.665–1 = 2.538), codominant model (GC vs. GG: *P* = 0.825, OR = 1.045, 95% CI: 0.709–1.540; CC vs. GG: *P* = 0.465, OR = 1.340, 95% CI: 0.611–2.939) and allelic model (*P* = 0.558, OR = 1.103, 95% CI: 0.795–1.531). Subgroup analysis on adults and Caucasians did not show any association between *IL-6*-174 G/C polymorphism and mortality. Nevertheless, subgroup analysis on non-adults manifested that *IL-6*-174 G/C polymorphism was associated with worse survival of septic patients in recessive model (*P* = 0.003, OR = 2.957, 95% CI: 1.442–6.066), co-dominant model (CC vs. GG: *P* = 0.005, OR = 3.221, 95% CI: 1.431–7.250) and allelic model (*P* = 0.015, OR = 1.631, 95% CI: 1.101–2.416).

### Publication bias and sensitivity analysis

No obvious publication bias was found in every section of this meta-analysis in Egger’s test. The results and graphs of Egger’s tests were shown in Table 2 and supplementary figures (see Additional file 2). The results of sensitivity analysis were shown in Table S2 (see Additional file 1). The results of meta-analysis on the association between *IL-6*-174 G/C polymorphism and the mortality of non-adult (CC vs. GG and allelic model) were changed after omitting certain studies. Additionally, some relevant studies might influence the results of study were also tested in our analyses. An analysis after omitting two studies including elder children from non-adults group was also made, the results being unchanged [32, 33] (see Additional file 1: Table S3). Moreover, an study was included in the meta-analysis by Gao et al. but excluded in our study [26]. Thus an analysis was carried out and proved that the exclusion of this study did not influence the results of our meta-analysis (see Additional file 1: Table S4).

### Trial sequential analysis

**Fig. 3:**
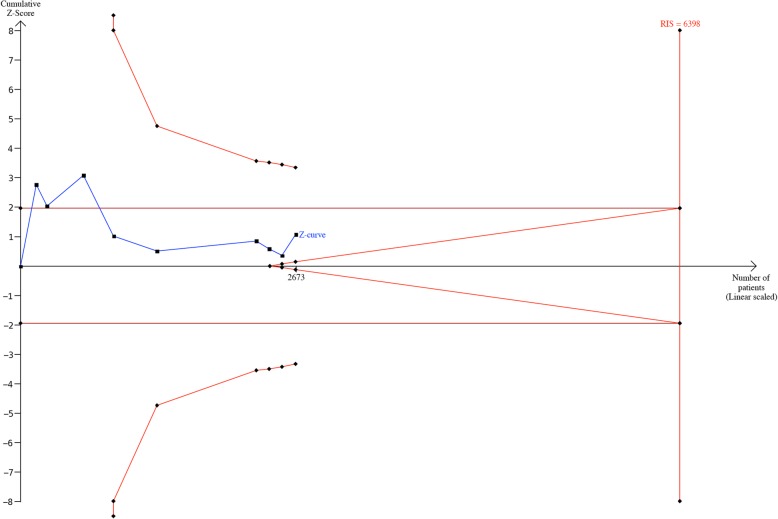
TSA of the analysis on the association between *IL-6*-174 G/C polymorphism and the risk of sepsis in non-adults under dominant model. A type I error of 5%, power of 80%, relative risk reduction of 20% were defined and control event proportion was an average of each included study. The vertical red line represents the required information size (sample size). The red curves represent the O’Brien-Fleming boundary and futility boundary. The blue line represents the cumulative Z-curve. RIS, required information size

In the meta-analysis on the risk of sepsis, TSA was conducted on analysis of overall population (dominant model and recessive model), non-adults (dominant model), adults (recessive model), Caucasians (recessive) and analysis involving healthy controls (allelic model) and Mendelian population (allelic model). Z-curves crossed the futility boundary and reached the required sample size in those analysis. The exception was the analysis on non-adult group, where Z-curve crossed neither conventional test boundary nor the futility boundary (Fig. 3). In the meta-analysis on the mortality of sepsis, TSA was conducted on analysis of overall population (allelic model), non-adults (recessive model, codominant model: CC vs. GG and allelic model), adults (allelic model) and Caucasians (allelic model). Although Z-curves crossed the conventional test boundary in the analyses on non-adults, they did neither cross O’Brien-Fleming boundary nor cross the RIS boundary (see Additional file 2). Z-curves in other TSAs crossed futility boundary and RIS boundary.

## Discussion

The association between *IL-6*-174 G/C polymorphism and the risk or mortality of sepsis has been widely studied, and previous meta-analyses have been conducted in 2008 and 2013. Five more studies have been published in recent years. Because of the controversial findings, an updated meta-analysis is necessary to bring some new insights into this topic. This study has the largest sample so far to address this issue. What’s more, trial sequential analysis was carried out in order to quantify the statistical reliability of the results. No association between *IL-6*-174 G/C polymorphism and the risk or mortality of sepsis in the overall population was found. This is similar to the finding by Gao et al. [9]. Heterogeneity in each study might be possible, which could arise from age, ethnicity or type of controls. However, subgroup analyses based on those factors did not present any association, either. Trial sequential analysis revealed that the results were reliable, except for the cases concerning non-adults group.

Located at promotor region of *IL-6* gene, − 174 G/C was found to be associated with the lower serum IL-6 level and lower inflammatory response subsequently [12, 27, 41]. Some previous studies reported the association between *IL-6*-174G/C polymorphism and the risk and mortality of sepsis [10–12, 16, 29, 31, 32, 34, 39, 40]. However, our results again failed to present any association. The reason could be: Firstly, the incidence and development of sepsis was involved in many factors such as gender, pathogens, gene polymorphism, environment effect and medical procedure. *IL-6*-174 G/C polymorphism might influence the transcriptional activity and lower the inflammatory reaction, it did not get the point that it could influence the development of sepsis nevertheless. Secondly, *IL-6*-174 G/C polymorphism might have an impact on the certain groups of people and pathological states, which were not within the subgroups of this study. Thirdly, *IL-6* had many promotor SNPs such as -597G/A, −572G/C, −373A_n_T_n_ and -174G/C, these polymorphic sites did not regulate the gene function independently [42]. And the influence of linkage disequilibrium could not be underestimated.

It’s noteworthy that C allele was associated with poor outcomes of sepsis in non-adults in recessive model, codominant model (CC vs. GG) and allelic model. However, TSA revealed that this result was not reliable as Z-curve crossed neither the conventional test boundary nor the futility boundary. Although many studies demonstrated C allele led to a lower *IL-6* expression, a study on neonates showed serum IL-6 level was higher in C carriers [43]. It was also found that IL-6 has an age-associated increase during systemic inflammation so that young persons should have a comparatively low IL-6 level during sepsis [44]. We hypothesized that higher IL-6 level induced by C allele could be more sensitive in very young persons and lead to aggravation of sepsis. This suggested different pathogenesis between non-adults and adults. It’s acknowledged that pediatric sepsis had different definitions, clinical presentations and management from adult sepsis. Maternal status also had an impact on newborns [45]. Thus the effect of *IL-6*-174 G/C polymorphism on the inflammatory process of non-adults might be promising in further studies.

Most included studies in our analysis were concerned with the Caucasians, rather than the Asians. Previous analysis also present that the frequency of *IL-6* -174G/C polymorphism were rare in Asian population that resided in Korea, Japan and Shandong province and Guangxi province of China [23, 24, 46, 47]. However, two studies included in our analysis reported possible association between *IL-6* -174G/C polymorphism and the risk of sepsis in the Chinese Han population in Henan province [11, 14]. This suggested that *IL-6* -174G/C polymorphism was distributed in Asians that resided in certain areas and might have an influence on the pathogenesis of sepsis.

As the types of controls were varied, it was considered that the status of controls could influence the results. In some studies, the case group was not comprised of septic patients only. Being compared to healthy controls and to mendelian population, the association with the risk of sepsis was not found, nevertheless. The case group of one study included a part of patients diagnosed as systemic inflammatory response syndrome (SIRS) or multiple organ disorder syndrome (MODS) [32]. And control group of one study consisted of ICU patients with no or mild sepsis [17] Because no exact number of the patients with or without sepsis was available, total group was included in the analysis. However, the results remained unchanged after omitting these studies in sensitivity analysis. In subgroup analysis of non-adults on the risk of sepsis, most of studies discussed age groups defined as infant or neonate. Only two studies addressed children under the age of 19 [32, 33]. After excluding these two studies including elder children, the results remained unchanged. In addition, one study that were included in the meta-analysis by Gao et al. was not included in ours, which was excluded due to limited information to obtain precise data and extreme small sample size of case group (4 patients with GG or GC genotype) [26]. And we have proven that the exclusion of this study did not affect the result of study.

Our study has two strengths. Firstly, to avoid our analysis to be biased from publication, database, inclusion criteria and language, strict criteria were laid out for enrolling studies and publications in the Chinese language were also searched. Publication bias was tested in Egger’s tests. Secondly, TSA was adopted to test the reliability of results and provide the research field which required more studies.

There were several limitations in this study. First, as the definition for sepsis changed, it was unlikely that the enrolled studies would have same definition because our analysis included studies within a large time range. Second, the majority of the objects studied were Caucasians, more studies were needed to further the study on other ethnicity groups. Third, three studies were not under Hardy-Weinberg equilibrium, although the results were not changed after omitting those studies. In addition, we could not get some details of the objects such as treatment and lifestyle, which prevented us from seeking more factors influencing the results.

## Conclusions

In conclusion, we did not find the evidence of the association between *IL-6*-174 G/C polymorphism and the risk or mortality of sepsis. Trial sequential analysis indicated that large sample size was needed to get a more reliable result for the association between *IL-6*-174 G/C polymorphism and the risk and mortality of sepsis in non-adults. More studies on the molecular mechanism and interactions between SNPs were needed.

## Additional files

Additional file 1: Supplementary tables. **Table S1**. Results of quality assessment of included studies using Newcastle-Ottawa Scale. **Table S2**. Summary of the studies with changed results after sensitivity analysis. **Table S3**. Results of meta-analysis on non-adults after excluding two studies discussing elder children. **Table S4**. Results of meta-analysis after including the study by Reiman. (DOCX 22 kb)

Additional file 2: Supplementary figures. The figures of pool analysis, egger’s test, sensitivity analysis and trial sequential analysis of each study were summarized. (DOCX 9463 kb)

## Abbreviations

CI: Confidential interval
CNKI: China National Knowledge Infrastructure
HWE: Hardy-Weinberg equilibrium
ICU: intensive care unit
IL-6: Interleukin-6
MODS: Multiple organ disorder syndrome
OR: Odd ratio
RIS: Required information size
SIRS: Systemic inflammatory response syndrome
SNP: Single nucleotide polymorphism
TSA: Trial sequential analysis
VLBW: Very low birth weight

## References

[1] Goulden R, Hoyle MC, Monis J (2018). qSOFA, SIRS and NEWS for predicting inhospital mortality and ICU admission in emergency admissions treated as sepsis. Emerg Med J.

[2] Singer M, Deutschman CS, Seymour CW (2016). The third international consensus definitions for Sepsis and septic shock (Sepsis-3). JAMA..

[3] Monneret G, Venet F (2016). Sepsis-induced immune alterations monitoring by flow cytometry as a promising tool for individualized therapy. Cytometry B Clin Cytom.

[4] Fan SL, Miller NS, Lee J, Remick DG (2016). Diagnosing sepsis - the role of laboratory medicine. Clin Chim Acta.

[5] Chauhan N, Tiwari S, Jain U (2017). Potential biomarkers for effective screening of neonatal sepsis infections: an overview. Microb Pathog.

[6] Tanaka T, Narazaki M, Kishimoto T (2016). Immunotherapeutic implications of IL-6 blockade for cytokine storm. Immunotherapy..

[7] Cole SW, Arevalo JM, Takahashi R (2010). Computational identification of gene-social environment interaction at the human IL6 locus. Proc Natl Acad Sci U S A.

[8] Chauhan M, McGuire W (2008). Interleukin-6 (−174C) polymorphism and the risk of sepsis in very low birth weight infants: meta-analysis. Arch Dis Child Fetal Neonatal Ed.

[9] Gao JW, Zhang AQ, Pan W (2015). Association between IL-6-174G/C polymorphism and the risk of sepsis and mortality: a systematic review and meta-analysis. PLoS One.

[10] Allam G, Alsulaimani AA, Alzaharani AK, Nasr A (2015). Neonatal infections in Saudi Arabia: association with cytokine gene polymorphisms. Cent Eur J Immunol.

[11] Mao ZR, Zhang SL, Feng B (2017). Association of IL-10 (−819T/C, −592A/C and -1082A/G) and IL-6-174G/C gene polymorphism and the risk of pneumonia-induced sepsis. Biomarkers..

[12] Feng B, Mao ZR, Pang K, Zhang SL, Li L (2015). Association of tumor necrosis factor alpha -308G/a and interleukin-6 -174G/C gene polymorphism with pneumonia-induced sepsis. J Crit Care.

[13] Lorente L, Martin MM, Perez-Cejas A, et al. Association between Interleukin-6 Promoter Polymorphism (−174 G/C), Serum Interleukin-6 Levels and Mortality in Severe Septic Patients. Int J Mol Sci. 2016;17(11).pii: E1861. 10.3390/ijms17111861 PMC5133861 27834822

[14] Jimenez-Sousa MA, Medrano LM, Liu P (2017). IL-6 rs1800795 polymorphism is associated with septic shock-related death in patients who underwent major surgery: a preliminary retrospective study. Ann Intensive Care.

[15] Zeng X, Zhang Y, Kwong JS (2015). The methodological quality assessment tools for preclinical and clinical studies, systematic review and meta-analysis, and clinical practice guideline: a systematic review. J Evid Based Med.

[16] Harding D, Dhamrait S, Millar A (2003). Montgomery H. Is interleukin-6 -174 genotype associated with the development of septicemia in preterm infants?. Pediatrics..

[17] Barber RC, Aragaki CC, Rivera-Chavez FA, Purdue GF, Hunt JL, Horton JW (2004). TLR4 and TNF-alpha polymorphisms are associated with an increased risk for severe sepsis following burn injury. J Med Genet.

[18] McDaniel DO, Hamilton J, Brock M (2007). Molecular analysis of inflammatory markers in trauma patients at risk of postinjury complications. J Trauma.

[19] Accardo Palumbo A, Forte GI, Pileri D (2012). Analysis of IL-6, IL-10 and IL-17 genetic polymorphisms as risk factors for sepsis development in burned patients. Burns..

[20] Carregaro F, Carta A, Cordeiro JA, Lobo SM, Silva EH, Leopoldino AM (2010). Polymorphisms IL10-819 and TLR-2 are potentially associated with sepsis in brazilian patients. Mem Inst Oswaldo Cruz.

[21] Gopel W, Hartel C, Ahrens P (2006). Interleukin-6-174-genotype, sepsis and cerebral injury in very low birth weight infants. Genes Immun.

[22] Wetterslev J, Jakobsen JC, Gluud C (2017). Trial Sequential Analysis in systematic. reviews with meta-analysis. BMC Med Res Methodol.

[23] Yang MX, Feng K, Zhang HX (2011). Correlation between IL-6 gene polymorphisms and sepsis of Chinese Han population in Henan province. Med J Chin PLA.

[24] Shimada T, Oda S, Sadahiro T (2011). Outcome prediction in sepsis combined use of genetic polymorphisms - a study in Japanese population. Cytokine..

[25] Shalhub S, Junker CE, Imahara SD, Mindrinos MN, Dissanaike S, O'Keefe GE (2009). Variation in the TLR4 gene influences the risk of organ failure and shock posttrauma: a cohort study. J Trauma.

[26] Reiman M, Kujari H, Ekholm E (2008). Interleukin-6 polymorphism is associated with chorioamnionitis and neonatal infections in preterm infants. J Pediatr.

[27] Tischendorf JJ, Yagmur E, Scholten D (2007). The interleukin-6 (IL6)-174 G/C promoter genotype is associated with the presence of septic shock and the ex vivo secretion of IL6. Int J Immunogenet.

[28] Schlüter B, Raufhake C, Erren M (2002). Effect of the interleukin-6 promoter polymorphism (−174G/C) on the incidence and outcome of sepsis. Crit Care Med.

[29] Balding J, Healy CM, Livingstone WJ (2003). Genomic polymorphic profiles in an Irish population with meningococcaemia: is it possible to predict severity and outcome of disease?. Genes Immun.

[30] Treszl A, Kocsis I, Szathmari M (2003). Genetic variants of TNF-[FC12] a, IL-1beta, IL-4 receptor [FC12]a-chain, IL-6 and IL-10 genes are not risk factors for sepsis in low-birth-weight infants. Biol Neonate.

[31] Ahrens P, Kattner E, Kohler B (2004). Mutations of genes involved in the innate immune system as predictors of sepsis in very low birth weight infants. Pediatr Res.

[32] Michalek J, Svetlikova P, Fedora M (2007). Interleukin-6 gene variants and the risk of sepsis development in children. Hum Immunol.

[33] Sipahi T, Pocan H, Akar N (2006). Effect of various genetic polymorphisms on the incidence and outcome of severe sepsis. Clin Appl Thromb Hemost.

[34] Baier RJ, Loggins J, Yanamandra K (2006). IL-10, IL-6 and CD14 polymorphisms and sepsis outcome in ventilated very low birth weight infants. BMC Med.

[35] Sabeļnikovs O, Ñikitina-Zaķe L, Vanags I (2008). Association of Interleukin 6 promoter polymorphism (−174G/C) with IL-6 level and outcome in severe Sepsis. Proceedings of the Latvian Academy of Sciences Section B Natural, Exact, and Applied Sciences.

[36] Sole-Violan J, de Castro F, Garcia-Laorden MI (2010). Genetic variability in the severity and outcome of community-acquired pneumonia. Respir Med.

[37] Davis SM, Clark EA, Nelson LT, Silver RM (2010). The association of innate immune response gene polymorphisms and puerperal group A streptococcal sepsis. Am J Obstet Gynecol.

[38] Watanabe E, Zehnbauer BA, Oda S, Sato Y, Hirasawa H, Buchman TG (2012). Tumor necrosis factor −308 polymorphism (rs1800629) is associated with mortality and ventilator duration in 1057 Caucasian patients. Cytokine..

[39] Martin-Loeches I, Sole-Violan J (2012). Rodriguez de Castro F, et al. variants at the promoter of the interleukin-6 gene are associated with severity and outcome of pneumococcal community-acquired pneumonia. Intensive Care Med.

[40] Abdel-Hady H, El-Naggar M, El-Nady G, Badr R, El-Daker M (2009). Genetic polymorphisms of IL-6–174 and IL-10–1082 in full term neonates with late onset blood stream infections. J Pediatr Infect Dis.

[41] Fishman D, Faulds G, Jeffery R (1998). The effect of novel polymorphisms in the interleukin-6 (IL-6) gene on IL-6 transcription and plasma IL-6 levels, and an association with systemic-onset juvenile chronic arthritis. J Clin Invest.

[42] Terry CF, Loukaci V, Green FR (2000). Cooperative influence of genetic polymorphisms on interleukin 6 transcriptional regulation. J Biol Chem.

[43] Kilpinen S, Hulkkonen J, Wang XY, Hurme M (2001). The promoter polymorphism of the interleukin-6 gene regulates interleukin-6 production in neonates but not in adults. Eur Cytokine Netw.

[44] Starr ME, Saito M, Evers BM, Saito H (2015). Age-associated increase in cytokine production during systemic inflammation—II: the role of IL-1β in age-dependent IL-6 upregulation in adipose tissue. J Gerontol Ser A Biol Med Sci.

[45] Prusakowski MK, Chen AP (2017). Pediatric Sepsis. Emerg Med Clin North Am.

[46] Lim CS, Zheng S, Kim YS (2002). The −174 G to C polymorphism of interleukin-6 gene is very rare in Koreans. Cytokine..

[47] Zhai R, Liu G, Yang C, Huang C, Wu C, Christiani DC (2001). The G to C polymorphism. at −174 of the interleukin-6 gene is rare in a Southern Chinese population. Pharmacogenetics.

