# Supplementary material for: The association between interleukin-6 gene -174G/C single nucleotide polymorphism and sepsis: an updated meta-analysis with trial sequential analysis
Source: BMC Med Genet. 2019 Feb 19;20:35. doi: 10.1186/s12881-019-0766-2 (PMC6379942; doi:10.1186/s12881-019-0766-2)
Supplement: Supplementary file 1 — Supplementary tables. Table S1. Results of quality assessment of included studies using Newcastle-Ottawa Scale. Table S2. Summary of the studies with changed results after sensitivity analysis. Table S3. Results of meta-analysis on non-adults after excluding two studies discussing elder children. Table S4. Results of meta-analysis after including the study by Reiman. (DOCX 22 kb) [file 12881_2019_766_MOESM1_ESM.docx]

| **Table S1 Results of quality assessment of included studies using Newcastle-Ottawa Scale** | | | | | | |  |  |  |
| --- | --- | --- | --- | --- | --- | --- | --- | --- | --- |
| First author | **Selection of cases** | | | | **Comparability** | **Exposure** | | | **Total** |
|  | Is the case definition adequate? | Representativeness of the cases | Selection of Controls | Definition of controls | Comparability of cases and controls on the basis of the design or analysis | Ascertainment of exposure | Same method of ascertainment for cases and controls | Non-Response rate |  |
| Schluter B (study on risk) | 1 | 1 | 1 | 1 | 1 | 1 | 1 | 1 | 8 |
| Schluter B (study on mortality) | 1 | 0 | 1 | 1 | 1 | 1 | 1 | 1 | 7 |
| Harding D | 1 | 1 | 0 | 1 | 1 | 1 | 1 | 1 | 7 |
| Balding J (study on risk) | 1 | 0 | 1 | 1 | 1 | 1 | 1 | 1 | 7 |
| Balding J (study on mortality) | 1 | 0 | 1 | 1 | 1 | 1 | 1 | 1 | 7 |
| Treszl A | 1 | 1 | 0 | 1 | 1 | 1 | 1 | 1 | 7 |
| Barber RC | 1 | 1 | 0 | 1 | 1 | 1 | 1 | 1 | 7 |
| Ahrens P | 1 | 1 | 0 | 1 | 1 | 1 | 1 | 1 | 7 |
| Michalek J | 0 | 1 | 1 | 1 | 1 | 1 | 1 | 1 | 7 |
| McDaniel DO (study on african american) | 1 | 1 | 0 | 1 | 1 | 1 | 1 | 1 | 7 |
| McDaniel DO (study on caucasian) | 1 | 1 | 0 | 1 | 1 | 1 | 1 | 1 | 7 |
| Sipahi T (study on risk) | 0 | 1 | 1 | 0 | 1 | 1 | 1 | 1 | 6 |
| Sipahi T (study on mortality) | 1 | 1 | 1 | 1 | 1 | 1 | 1 | 1 | 8 |
| Baier RJ (study on risk-afriacan american) | 1 | 1 | 0 | 1 | 1 | 1 | 1 | 1 | 7 |
| Baier RJ (study on risk-caucasian) | 1 | 1 | 0 | 1 | 1 | 1 | 1 | 1 | 7 |
| Baier RJ (study on mortality-afriacan american) | 1 | 1 | 1 | 1 | 1 | 1 | 1 | 1 | 8 |
| Baier RJ (study on mortality-afriacan american) | 1 | 1 | 1 | 1 | 1 | 1 | 1 | 1 | 8 |
| Göpel W | 1 | 1 | 0 | 1 | 1 | 1 | 1 | 1 | 7 |
| Sabeinikovs O | 1 | 1 | 1 | 1 | 1 | 1 | 1 | 1 | 8 |
| Abdel-Hady H (study on risk) | 1 | 0 | 1 | 1 | 1 | 1 | 1 | 1 | 7 |
| Abdel-Hady H (study on mortality) | 1 | 0 | 1 | 1 | 1 | 1 | 1 | 1 | 7 |
| Solé-Violán J | 1 | 1 | 0 | 1 | 1 | 1 | 1 | 1 | 7 |
| Davis SM | 1 | 0 | 1 | 1 | 1 | 1 | 1 | 1 | 7 |
| Carregaro F | 1 | 0 | 1 | 1 | 1 | 1 | 1 | 1 | 7 |
| Accardo Palumbo A | 1 | 1 | 0 | 1 | 1 | 1 | 1 | 1 | 7 |
| Watanabe E | 1 | 1 | 1 | 1 | 1 | 1 | 1 | 1 | 8 |
| Martín-Loeches I (study on risk) | 1 | 0 | 1 | 1 | 1 | 1 | 1 | 1 | 7 |
| Martín-Loeches I (study on monrtality) | 1 | 0 | 1 | 1 | 1 | 1 | 1 | 1 | 7 |
| Feng B | 1 | 0 | 1 | 0 | 1 | 1 | 1 | 1 | 6 |
| Allam G | 1 | 0 | 1 | 1 | 1 | 1 | 1 | 1 | 7 |
| Lorente L | 1 | 1 | 1 | 1 | 1 | 1 | 1 | 1 | 8 |
| Mao ZR | 1 | 1 | 1 | 1 | 1 | 1 | 1 | 1 | 8 |
| Jimenez-Sousa MA | 1 | 1 | 1 | 1 | 1 | 1 | 1 | 1 | 8 |

| **Table S2 Summary of the studies with changed results after sensitivity analysis** | | |
| --- | --- | --- |
| First author of the omitted study | Involved analysis | Effect |
| **The relationship between *IL-6* -174G/C polymorphism and the mortality of sepsis** | | |
| Sabeinikovs O | subgroup analysis on non-adult(CC vs. GG) | *P* = 0.07, OR = 2.804, 95%CI: 0.921-8.541 |
|  | subgroup analysis on non-adult(allelic model) | *P* = 0.168, OR = 1.479, 95%CI: 0.848-2.578 |
| Abdel-Hady H | subgroup analysis on non-adult(CC vs. GG) | *P* = 0.057, OR = 2.456, 95%CI: 0.975-6.188 |
|  | subgroup analysis on non-adult(allelic model) | *P* = 0.136, OR = 1.398, 95%CI: 0.900-2.171 |

| **Table S3 Results of meta-analysis on non-adults after excluding two studies discussing elder children** | | | | | | |
| --- | --- | --- | --- | --- | --- | --- |
|  | **Meta-analysis** | |  | **Heterogeneity test** | | **Egger's test** |
|  | **no. of studies** | **OR (95% CI)** | ***P* value** | ***I^2^*** | ***P* value** | ***P* value** |
| **The association between *IL-6* -174G/C polymorphism and the risk of sepsis^a^** | | | | | | |
| GC+CC vs GG | 7 | 0.745(0.415-1.336) | 0.323 | 82.30% | <0.001 | 0.132 |
| CC vs. GC+GG | 6 | 0.943(0.665-1.338) | 0.774 | 44.40% | 0.109 | 0.728 |
| GC vs. GG | 6 | 0.825(0.451-1.511) | 0.534 | 78.70% | <0.001 | 0.198 |
| CC vs.GG | 6 | 0.792(0.335-1.874) | 0.595 | 74.40% | 0.002 | 0.581 |
| C vs. G | 6 | 0.874(0.541-1.410) | 0.581 | 83.80% | <0.001 | 0.423 |
| ^a^ The results remained unchanged in sensitivity analyses and trial sequential analysis indicated more studies were necessary to reach required sample size. | | | | | | |

| **Table S4. Results of meta-analysis after including the study by Reiman.** | | | | | | |
| --- | --- | --- | --- | --- | --- | --- |
|  | **Meta-analysis** | | | **Heterogeneity test** | | **Egger's test** |
|  | **no. of studies** | **OR (95% CI)** | ***P* value** | ***I^2^*** | ***P* value** | ***P* value** |
| **The association between *IL-6* -174G/C polymorphism and the risk of sepsis** | | | | | | |
| **Overall** |  |  |  |  |  |  |
| CC vs. GC+GG | 19 | 1.060(0.769-1.462) | 0.723 | 74.70% | <0.001 | 0.699 |
| **Non-adult** | |  |  |  |  |  |
| CC vs. GC+GG | 9 | 0.785(0.614-1.003) | 0.053 | 41.50% | 0.101 | 0.337 |
| **Caucasian** | |  |  |  |  |  |
| CC vs. GC+GG | 18 | 0.892(0.771-1.033) | 0.127 | 6.70% | 0.376 | 0.712 |
