# Supplementary material for: The association between interleukin-6 gene -174G/C single nucleotide polymorphism and sepsis: an updated meta-analysis with trial sequential analysis
Source: BMC Med Genet. 2019 Feb 19;20:35. doi: 10.1186/s12881-019-0766-2 (PMC6379942; doi:10.1186/s12881-019-0766-2)
Supplement: Supplementary file 2 — Supplementary figures. The figures of pool analysis, egger’s test, sensitivity analysis and trial sequential analysis of each study were summarized. (DOCX 9463 kb) [file 12881_2019_766_MOESM2_ESM.docx]

**The association between interleukin-6 gene -174G/C single nucleotide polymorphism and** **sepsis: An updated meta-analysis with trial sequential analysis**

Yao Chen, Yanyan Hu and Zhenju Song

**Supplementary figures**

**Interpretations of figures**

**Forest plots:**

The horizontal line represents the 95% confidential interval. And its length shows the range of the confidential interval and the size of the square in the middle shows the weight of the study. The diamond (and broken line) represents the overall summary estimate, with confidence interval given by its width. The unbroken vertical line is at the null value (OR = 1.0). CI, confidence interval.

**Figure of Egger’s linear regression test:**

Each point represents a separate study for the indicated association. The X-axis represents the precision and the Y-axis represents the standard normal deviate of effect estimate. The vertical line represents the 95% confidential interval and its length shows the range of the confidential interval. Once 0 was within the range of 95% CI in each regression test, no obvious publication bias existed.

**Figure of sensitivity analysis:**

Each horizontal line represents the 95% confidential interval after excluding omitted study. And its length shows the range of the confidential interval. The diamond (and broken line) represents the overall summary estimate. The vertical line in the middle and both sides represent the overall summary estimate and 95% confidential interval of origin meta-analysis, respectively. CI, confidence interval.

**Figure of trial sequential analysis:**

The vertical red line represents the required information size (sample size). The red curves represent the O’Brien-Fleming boundary and futility boundary. The blue line represents the cumulative Z-curve. RIS: required information size.

**Table of contents**

**The association between *IL-6* -174G/C polymorphism and the risk of sepsis**

| **Overall** | **Page** |
| --- | --- |
| GC+CC vs GG | 5 |
| CC vs. GC+GG | 6 |
| GC vs. GG | 7 |
| CC vs.GG | 8 |
| C vs. G | 9 |
| **Non-adult** |  |
| GC+CC vs GG | 10 |
| CC vs. GC+GG | 11 |
| GC vs. GG | 12 |
| CC vs.GG | 13 |
| C vs. G | 14 |
| **Adult** |  |
| GC+CC vs GG | 15 |
| CC vs. GC+GG | 16 |
| GC vs. GG | 17 |
| CC vs.GG | 18 |
| C vs. G | 19 |
| **Caucasian** |  |
| GC+CC vs GG | 20 |
| CC vs. GC+GG | 21 |
| GC vs. GG | 22 |
| CC vs.GG | 23 |
| C vs. G | 24 |
| **Healthy control** |  |
| GC+CC vs GG | 25 |
| CC vs. GC+GG | 26 |
| GC vs. GG | 27 |
| CC vs.GG | 28 |
| C vs. G | 29 |
| **Mendelian population** |  |
| GC+CC vs GG | 30 |
| CC vs. GC+GG | 31 |
| GC vs. GG | 32 |
| CC vs.GG | 33 |
| C vs. G | 34 |

**The association between *IL-6* -174G/C polymorphism and the mortality of sepsis**

| **Overall** | **Page** |
| --- | --- |
| GC+CC vs GG | 35 |
| CC vs. GC+GG | 36 |
| GC vs. GG | 37 |
| CC vs.GG | 38 |
| C vs. G | 39 |
| **Non-adult** |  |
| GC+CC vs GG | 40 |
| CC vs. GC+GG | 41 |
| GC vs. GG | 42 |
| CC vs.GG | 43 |
| C vs. G | 44 |
| **Adult** |  |
| GC+CC vs GG | 45 |
| CC vs. GC+GG | 46 |
| GC vs. GG | 47 |
| CC vs.GG | 48 |
| C vs. G | 49 |
| **Caucasian** |  |
| GC+CC vs GG | 50 |
| CC vs. GC+GG | 51 |
| GC vs. GG | 52 |
| CC vs.GG | 53 |
| C vs. G | 54 |

**The association between *IL-6*-174 G/C polymorphism and the risk of sepsis**

1. Overall analysis

1.1 dominant model

(1) Forest plots

see Figure 2

(2) Figure of Egger’s linear regression test


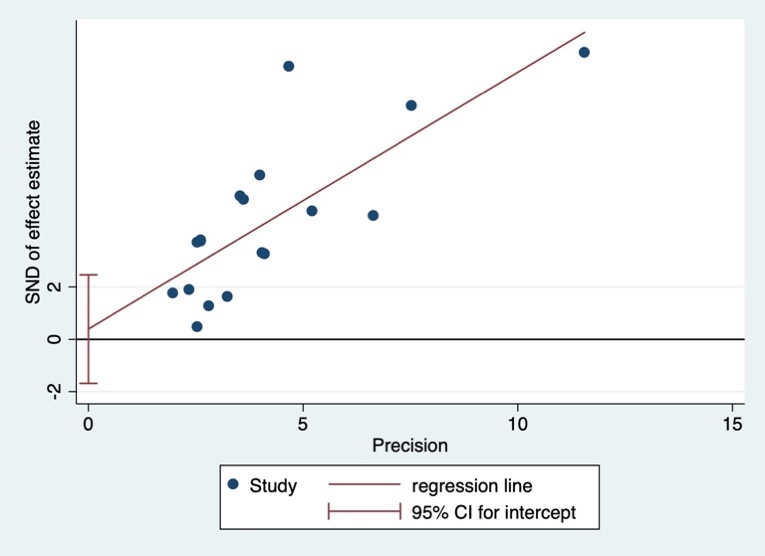


(3) Figure of sensitivity analysis


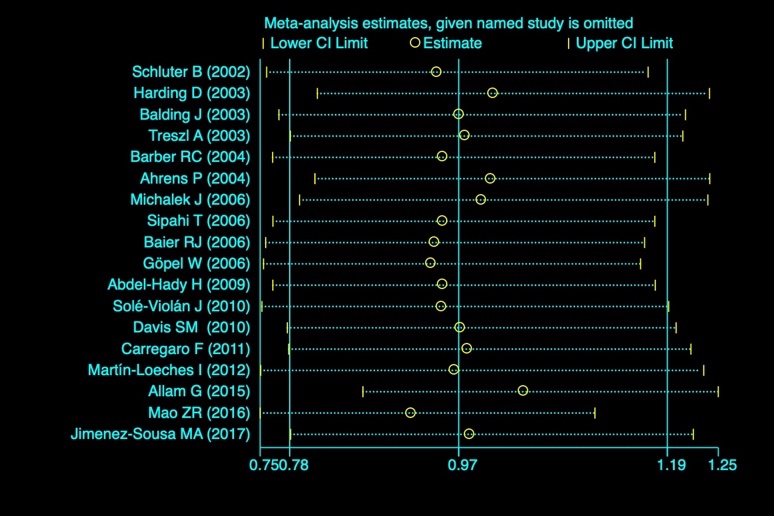


（4）Figure of trial sequential analysis


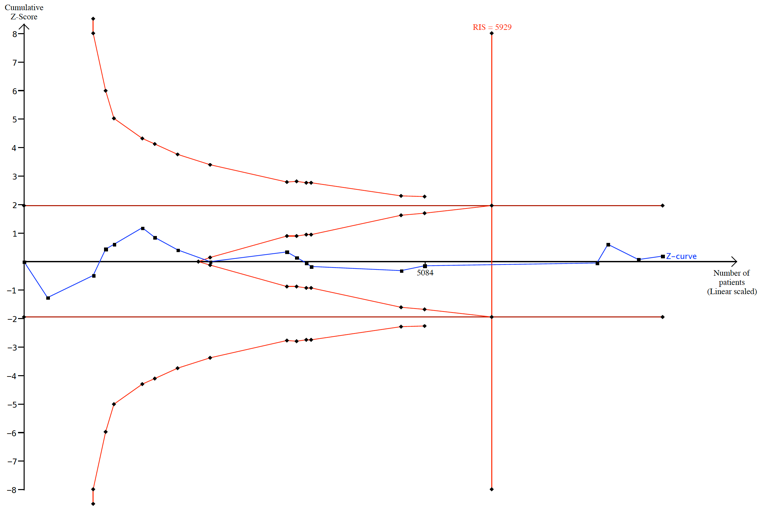


1.2 recessive model

(1) Forest plots


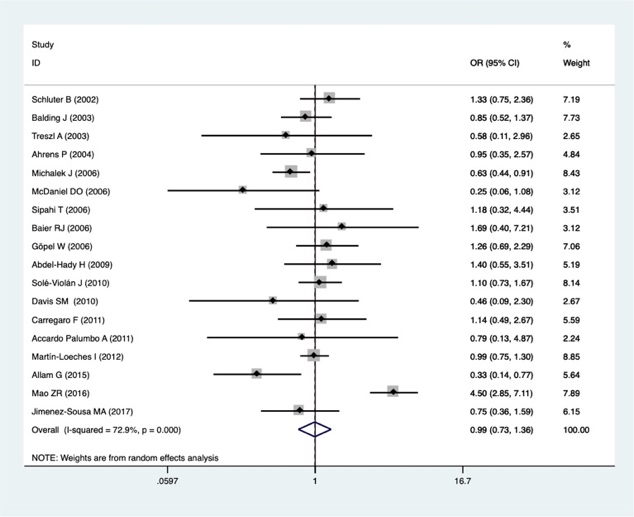


(2) Figure of Egger’s linear regression test


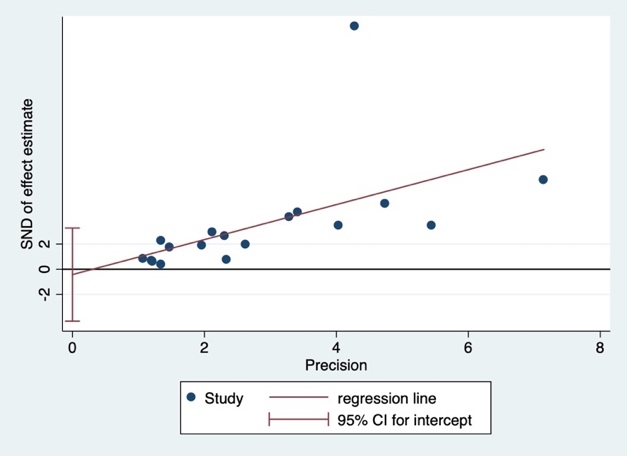


(3) Figure of sensitivity analysis


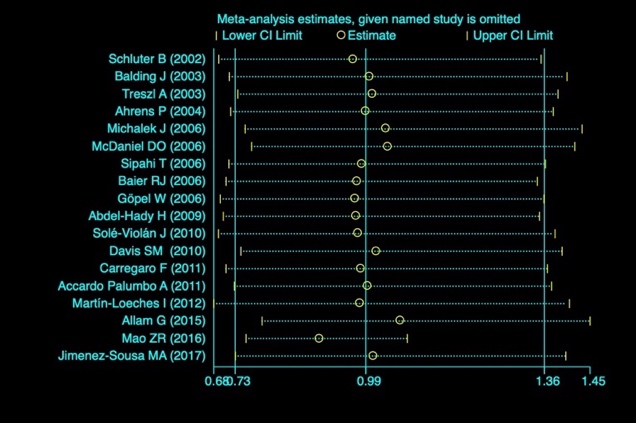


(4) Figure of trial sequential analysis


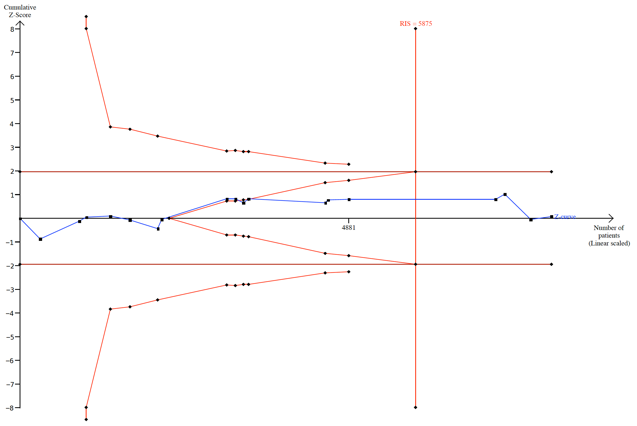


1.3 codominant model: GC vs. GG

(1) Forest plots


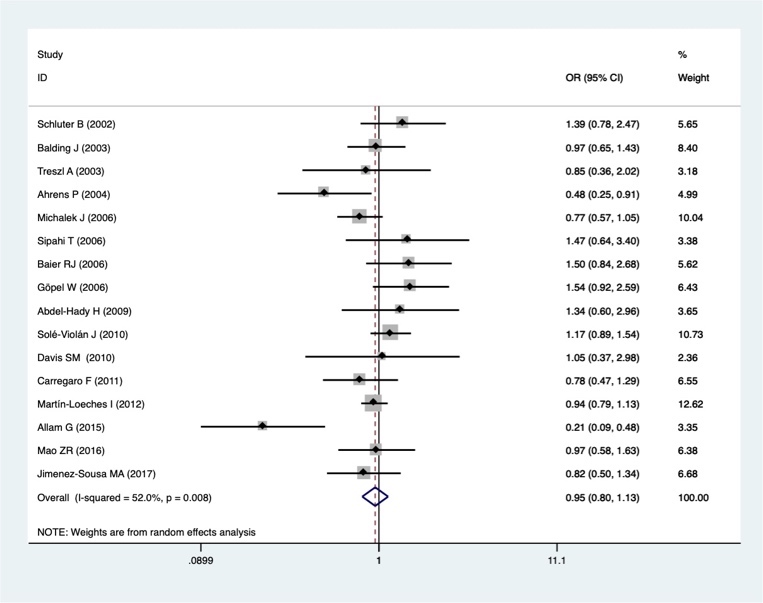


(2) Figure of Egger’s linear regression test


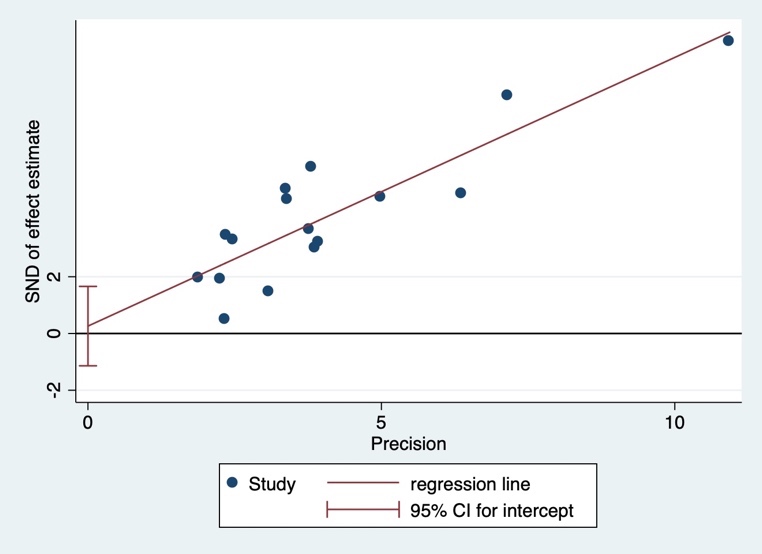


(3) Figure of sensitivity analysis


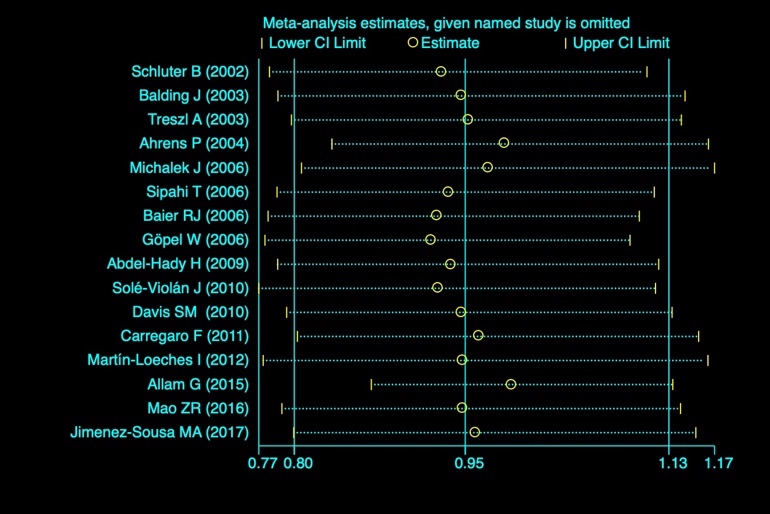


1.4 codominant model: CC vs. GG

(1) Forest plots


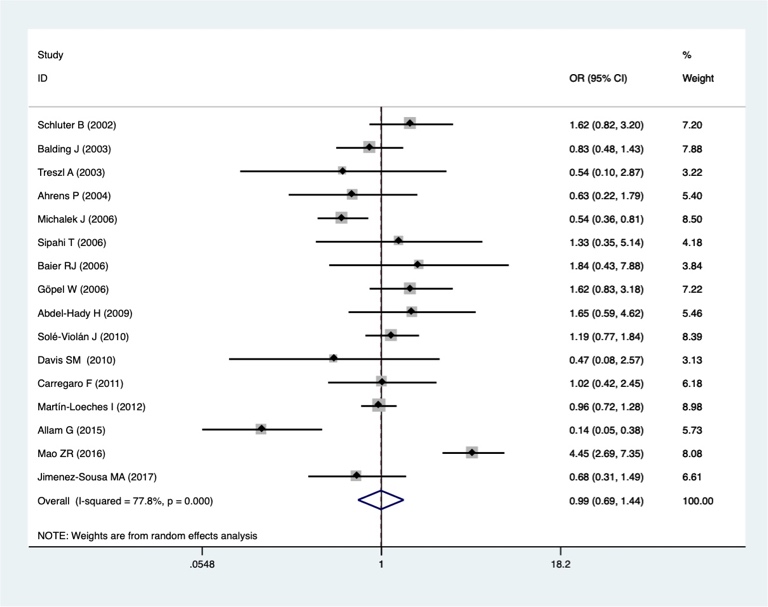


(2) Figure of Egger’s linear regression test


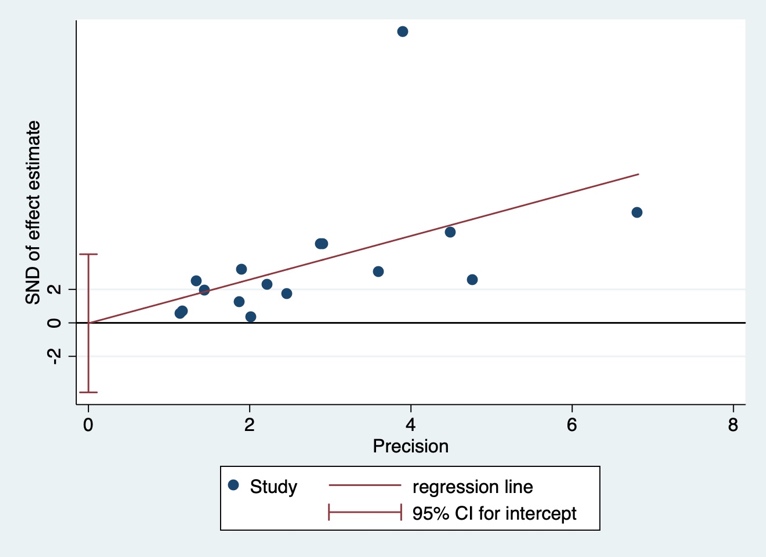


(3) Figure of sensitivity analysis


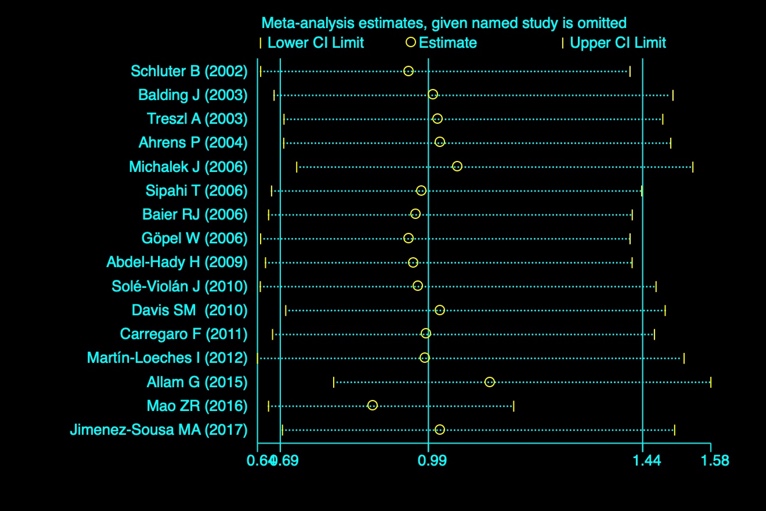


1.5 allelic model

(1) Forest plots


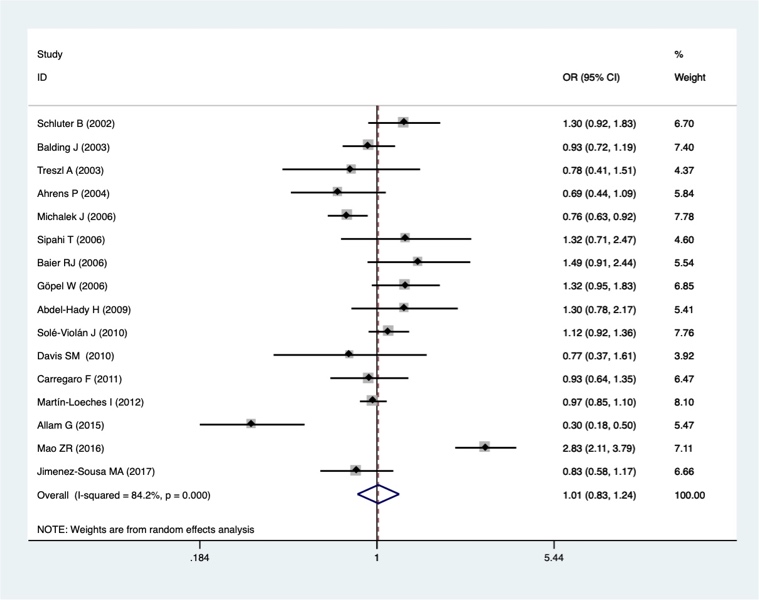


(2) Figure of Egger’s linear regression test


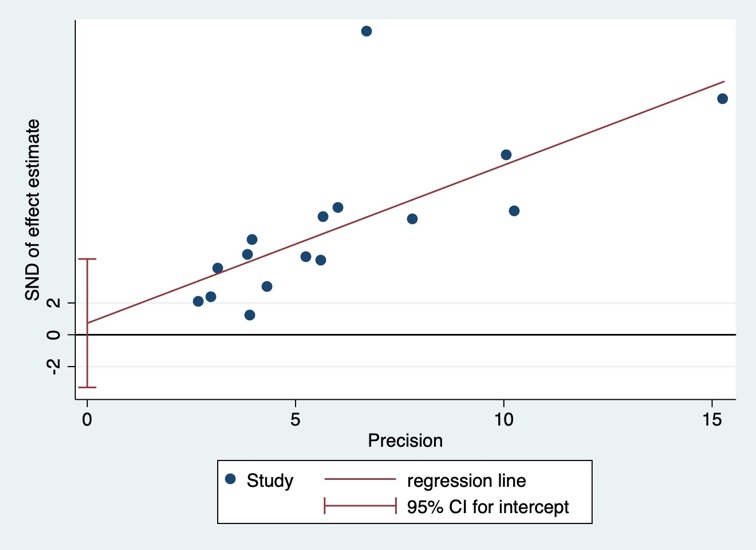


(3) Figure of sensitivity analysis


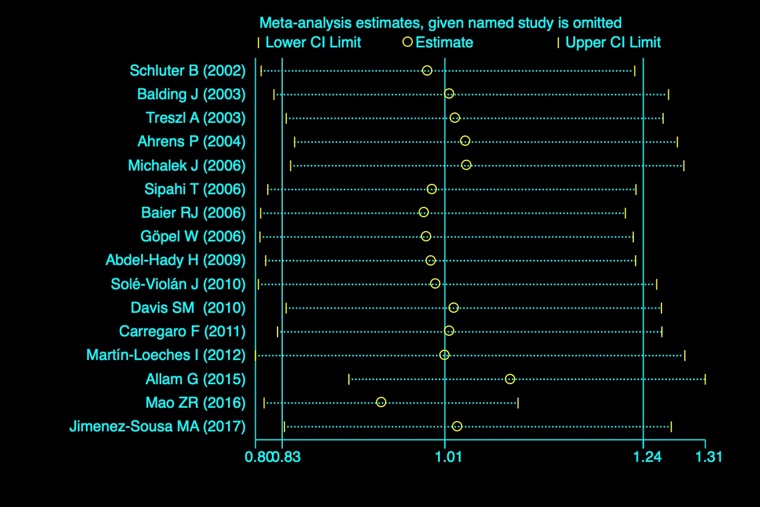


2. Non-adult

2.1 dominant model

(1) Forest plots


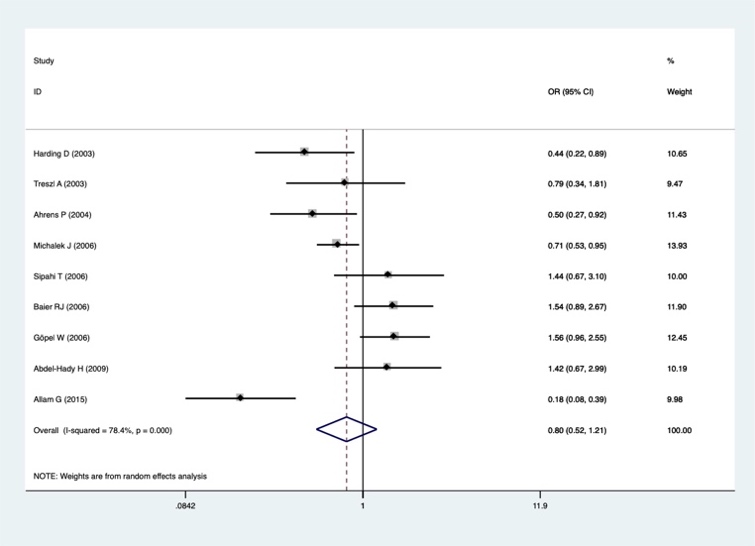


(2) Figure of Egger’s linear regression test


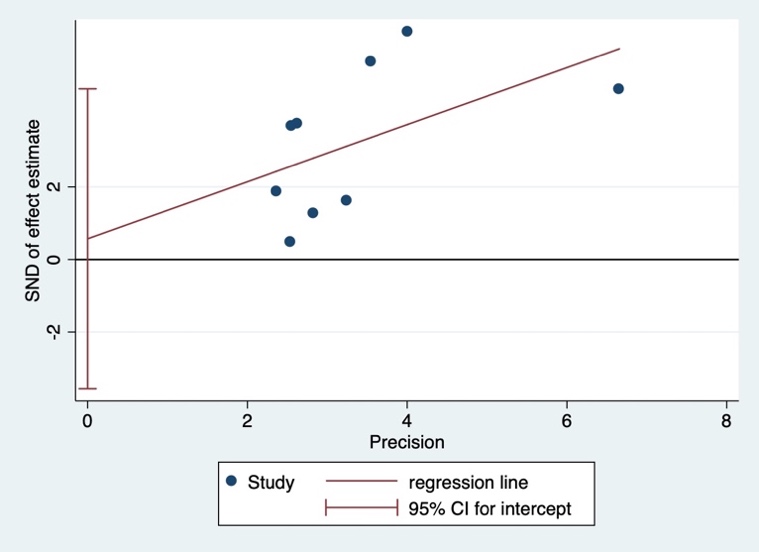


(3) Figure of sensitivity analysis


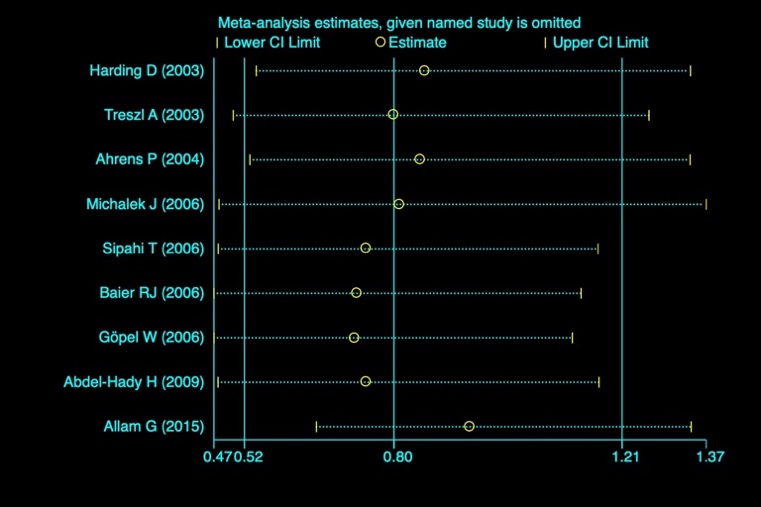


(4) Figure of trial sequential analysis

See Figure 3

2.2 recessive model

(1) Forest plots


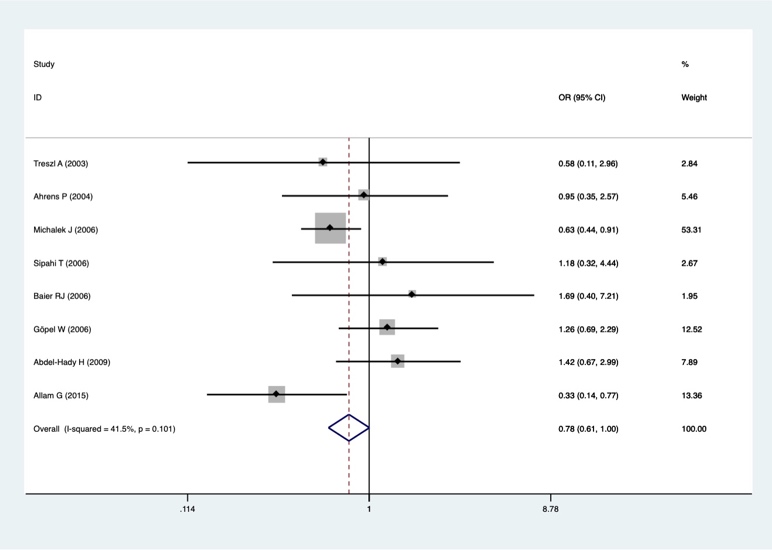


(2) Figure of Egger’s linear regression test


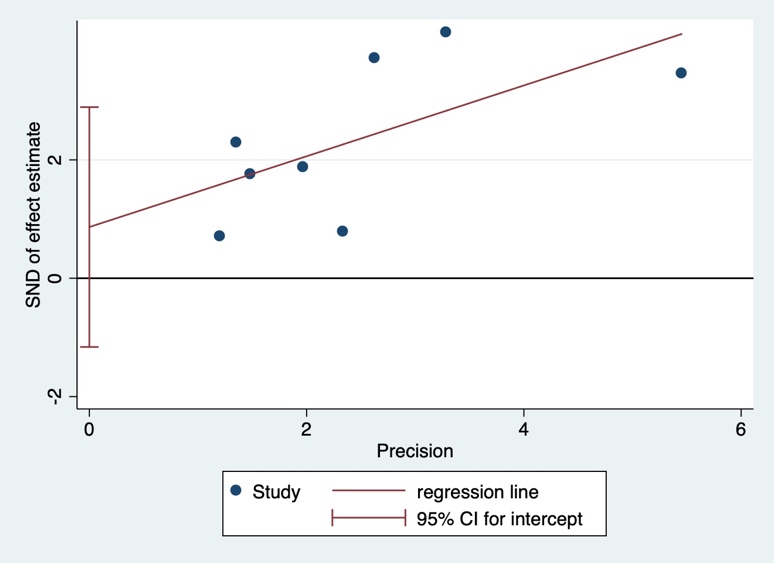


(3) Figure of sensitivity analysis


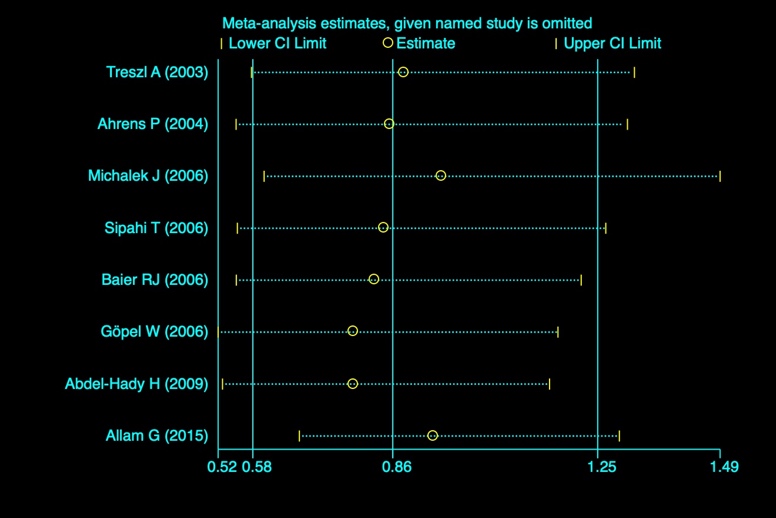


2.3 codominant model: GC vs. GG

(1) Forest plots


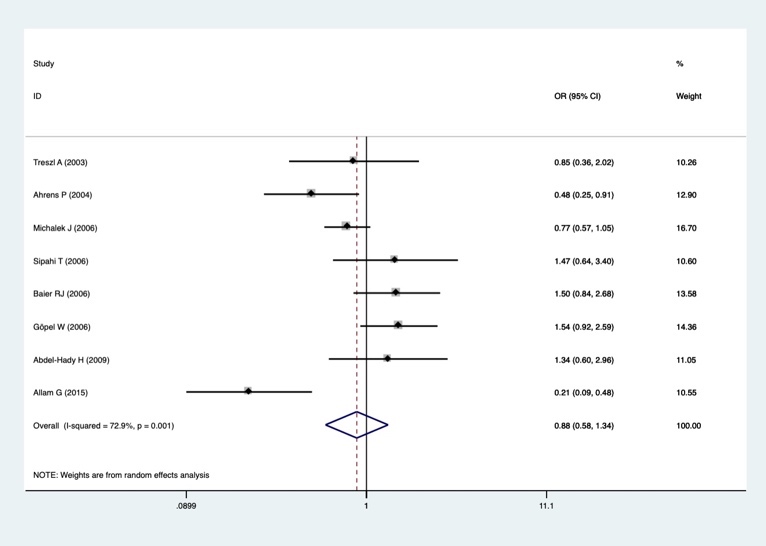


(2) Figure of Egger’s linear regression test


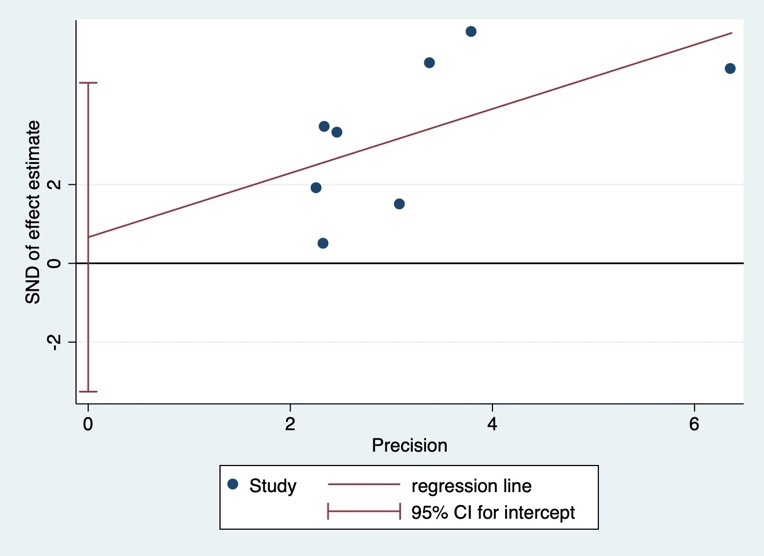


(3) Figure of sensitivity analysis


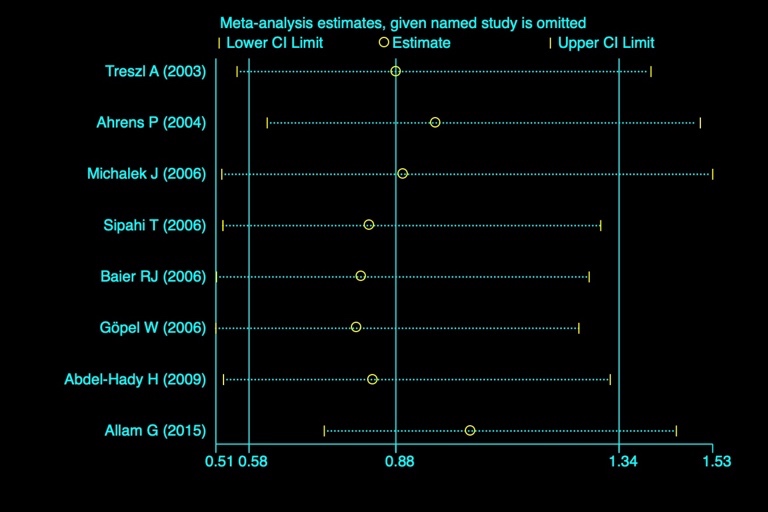


2.4 codominant model: CC vs. GG

(1) Forest plots


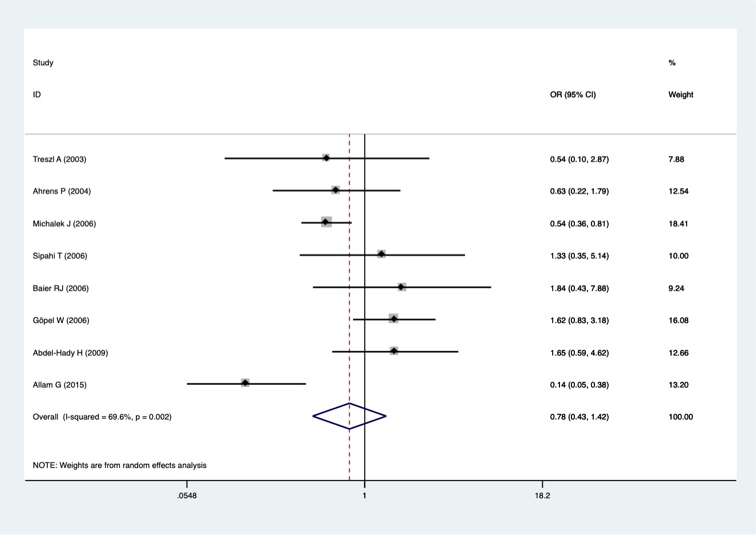


(2) Figure of Egger’s linear regression test


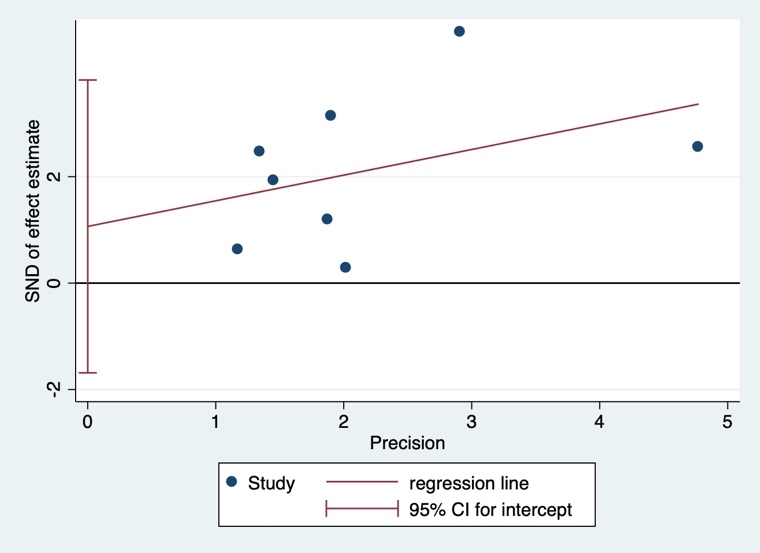


(3) Figure of sensitivity analysis


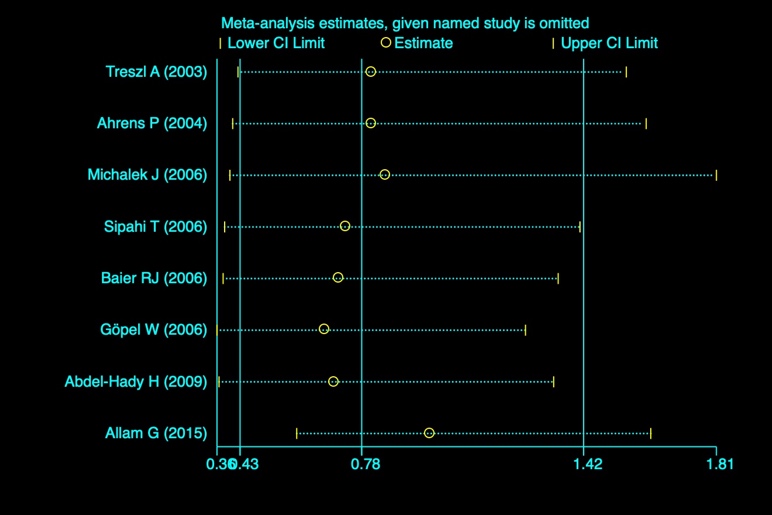


2.5 allelic model

(1) Forest plots


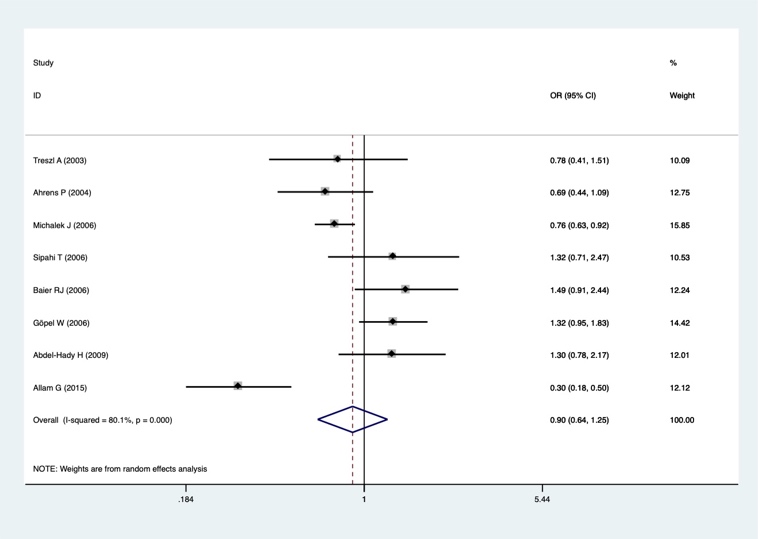


(2) Figure of Egger’s linear regression test


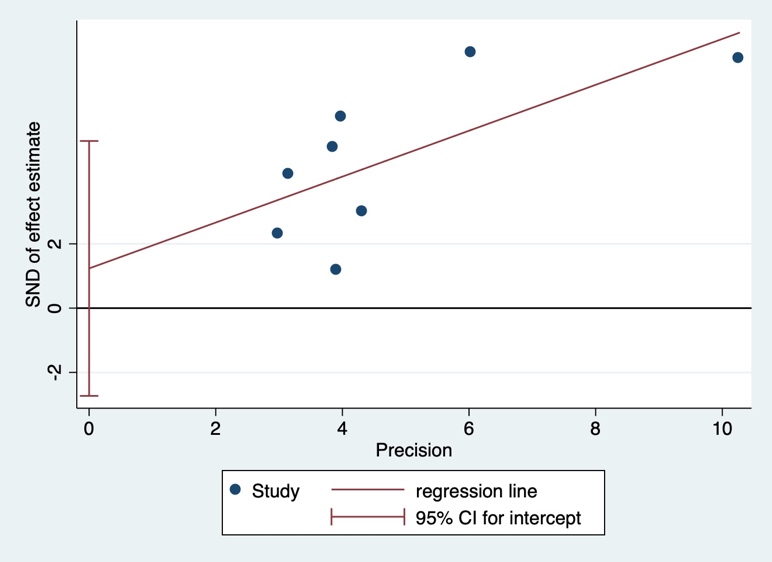


(3) Figure of sensitivity analysis


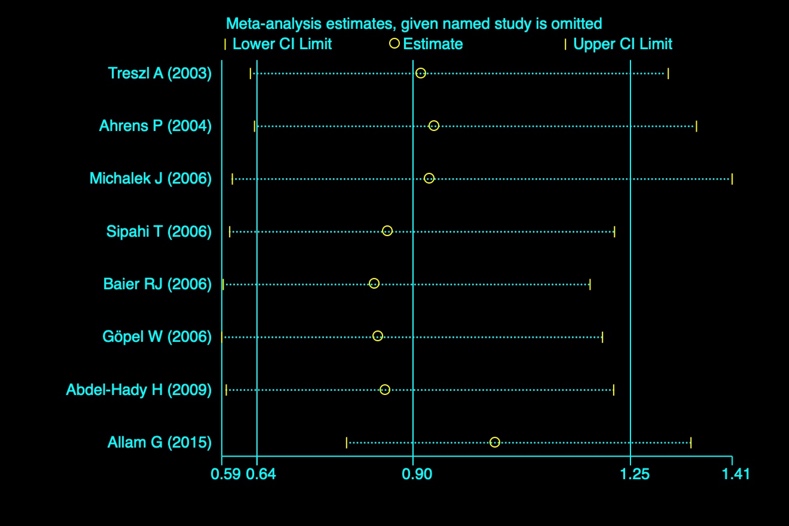


3. Adult

3.1 dominant model

(1) Forest plots


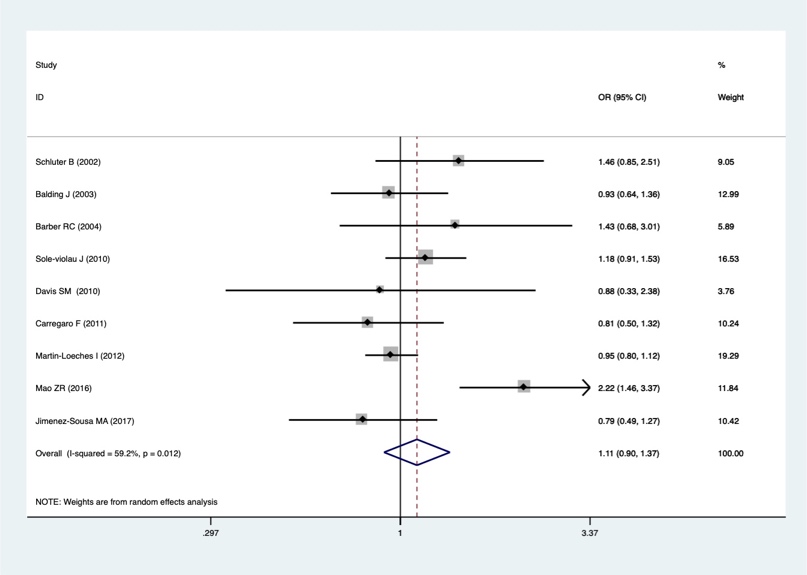


(2) Figure of Egger’s linear regression test


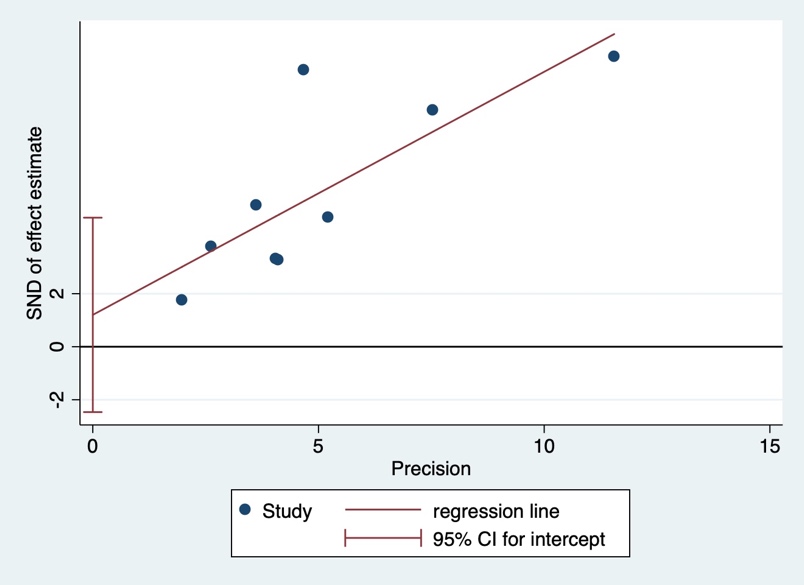


(3) Figure of sensitivity analysis


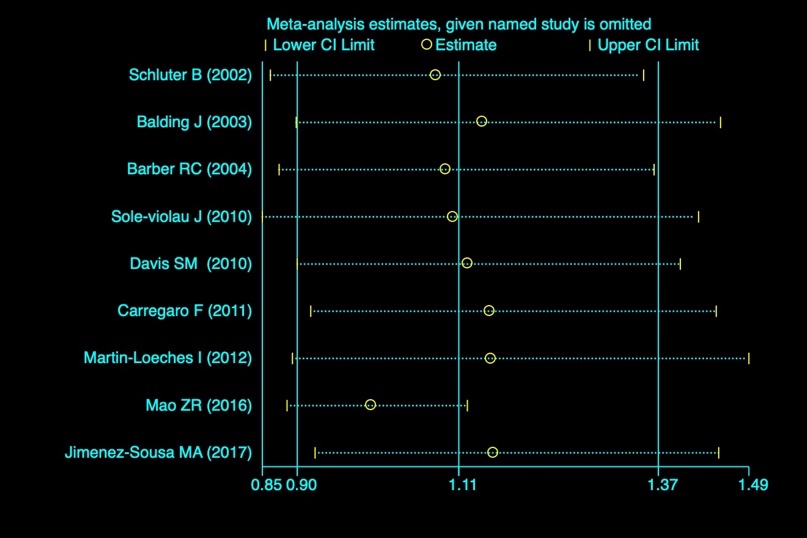


3.2 recessive model

(1) Forest plots


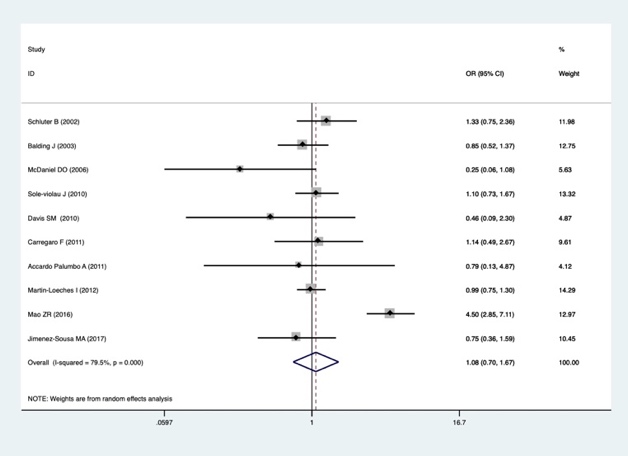


(2) Figure of Egger’s linear regression test


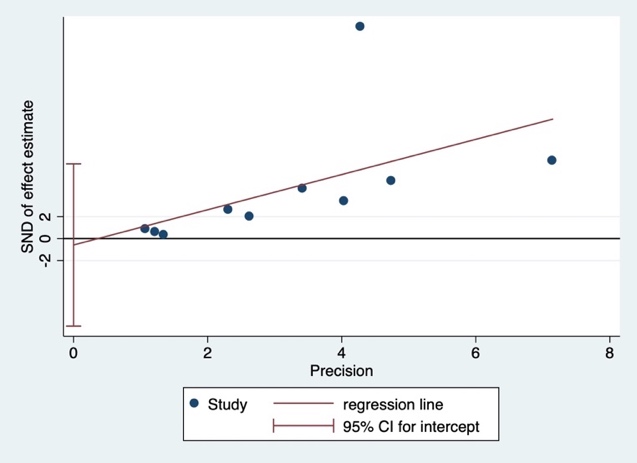


(3) Figure of sensitivity analysis


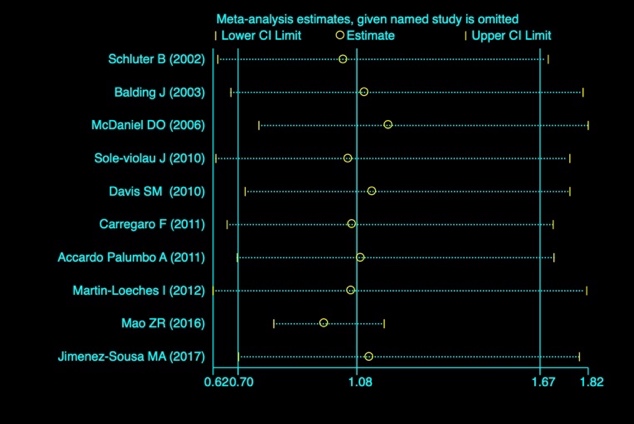


(4) Figure of trial sequential analysis


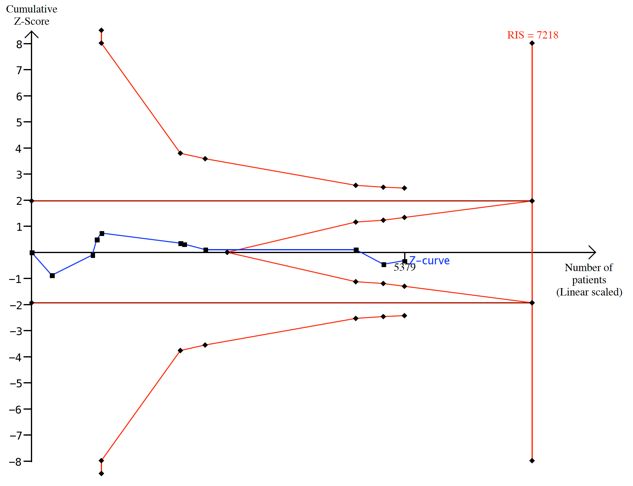


3.3 codominant model GC vs. GG

(1) Forest plots


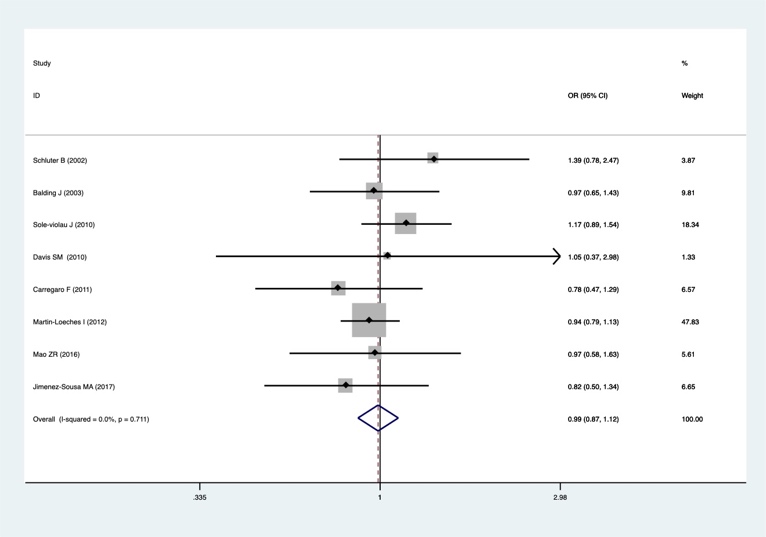


(2) Figure of Egger’s linear regression test


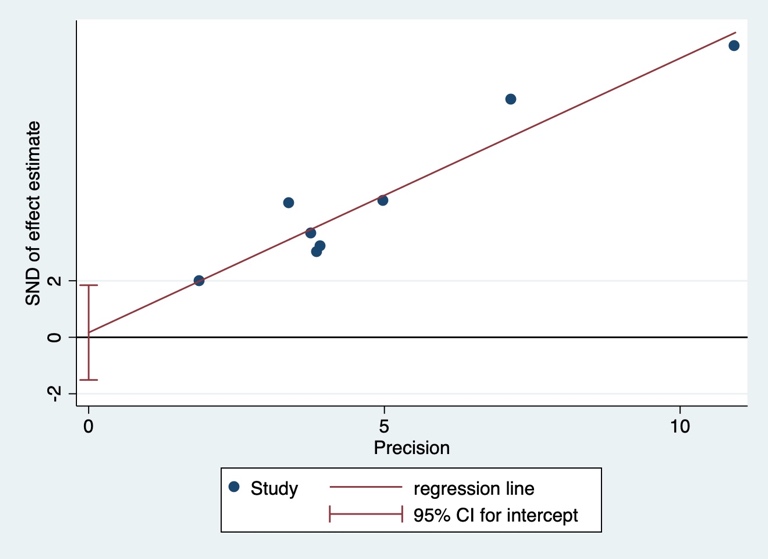


(3) Figure of sensitivity analysis


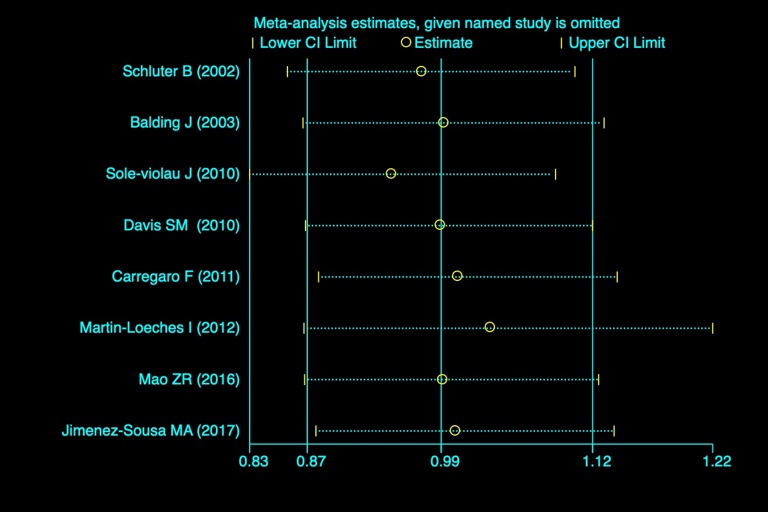


3.4 codominant model CC vs. GG

(1) Forest plots


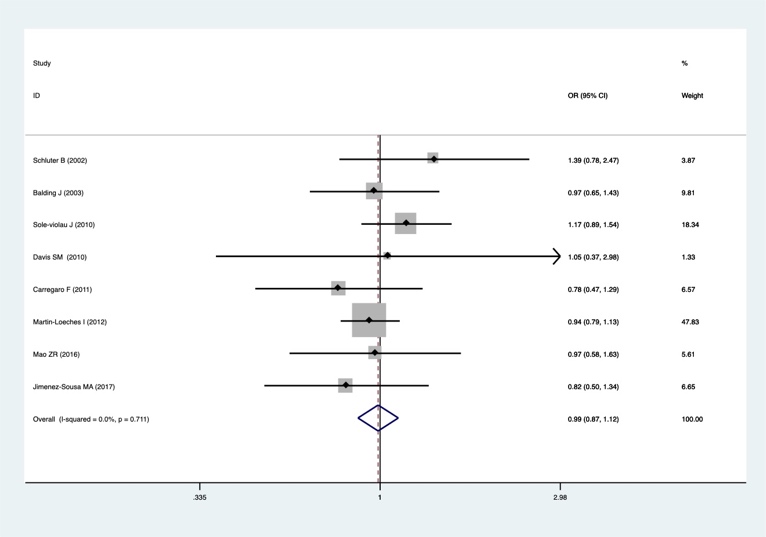


(2) Figure of Egger’s linear regression test


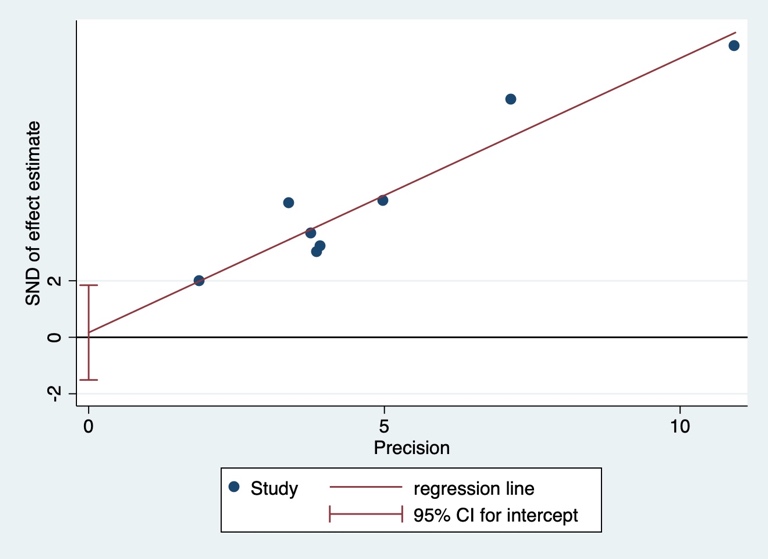


(3) Figure of sensitivity analysis


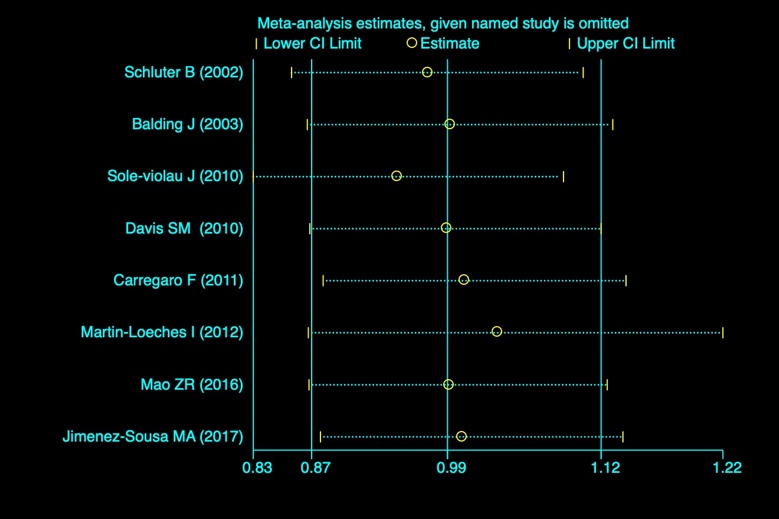


3.5 allelic model

(1) Forest plots


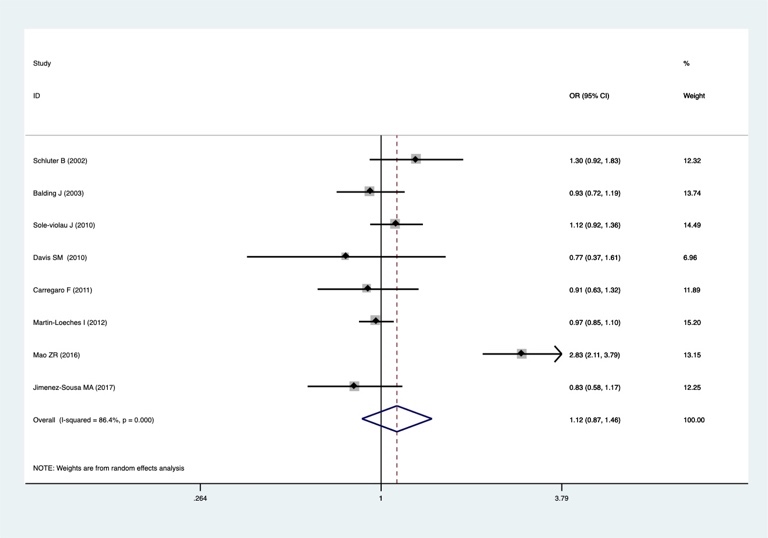


(2) Figure of Egger’s linear regression test


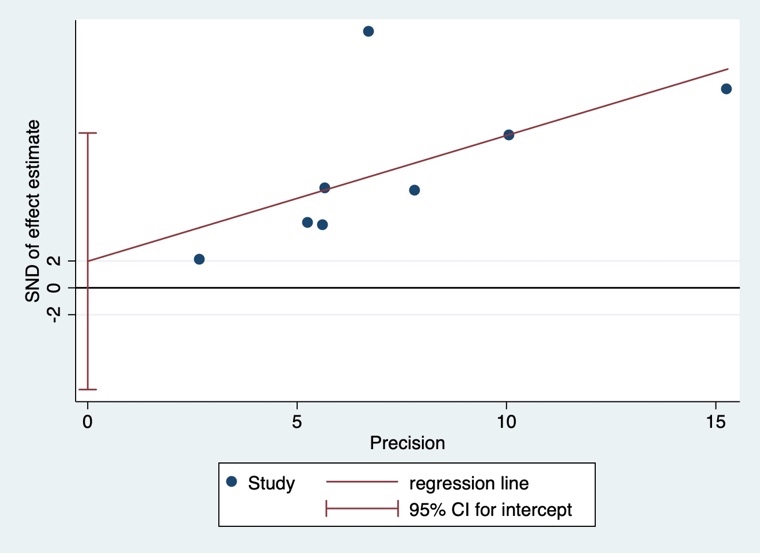


(3) Figure of sensitivity analysis


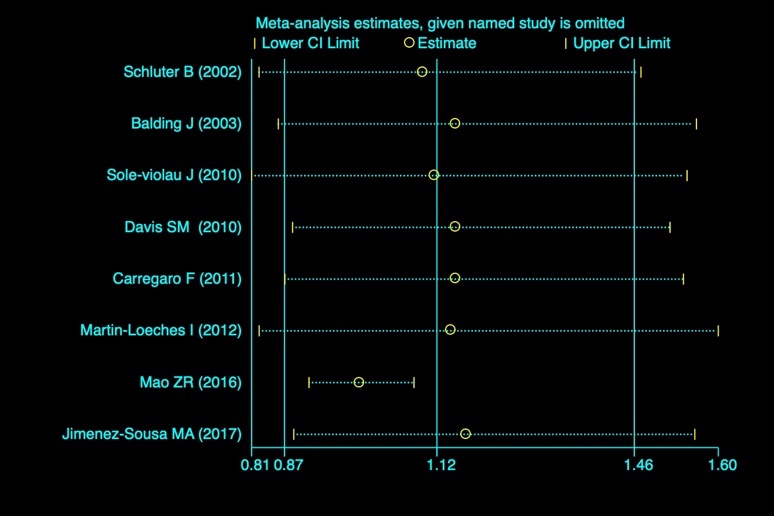


4. Caucasian

4.1 dominant model

(1) Forest plots


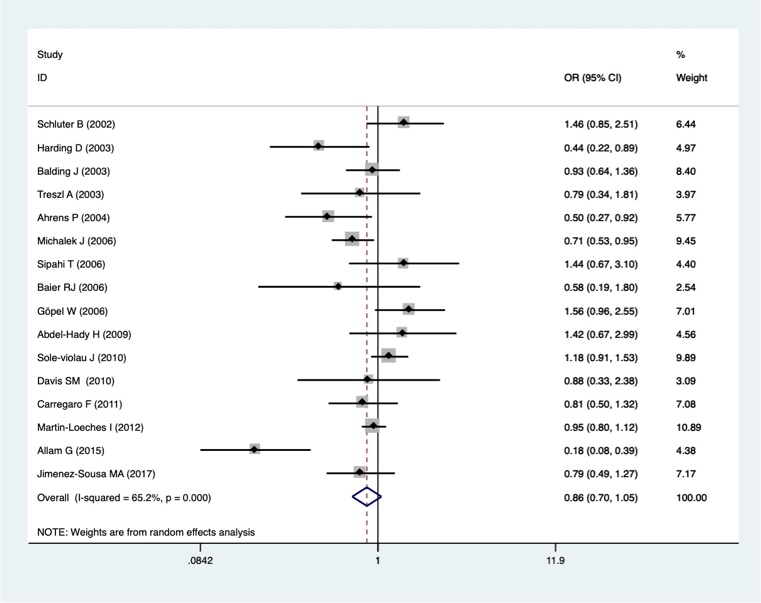


(2) Figure of Egger’s linear regression test


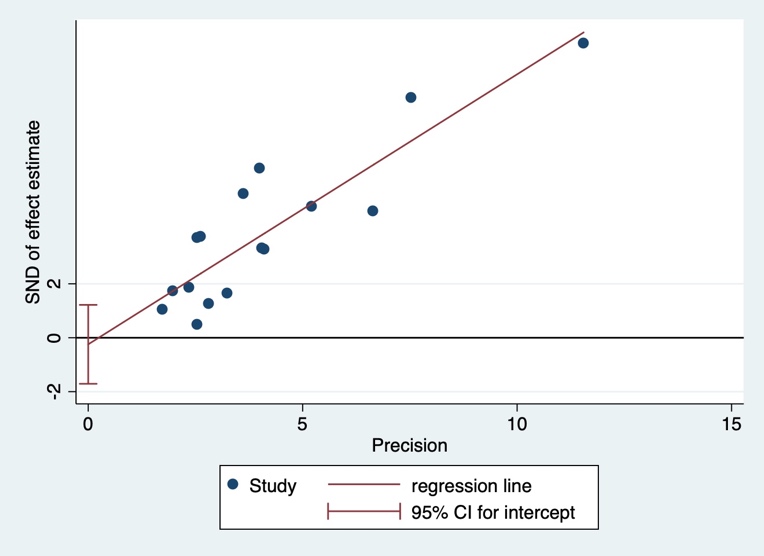


(3) Figure of sensitivity analysis


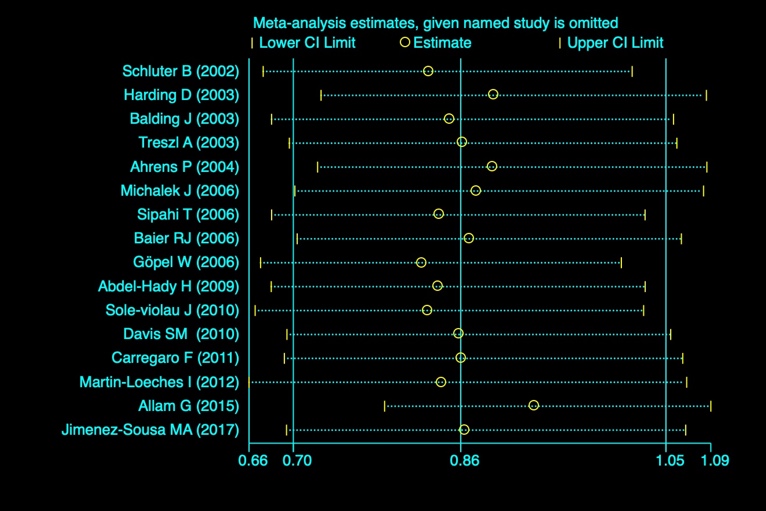


4.2 recessive model

(1) Forest plots


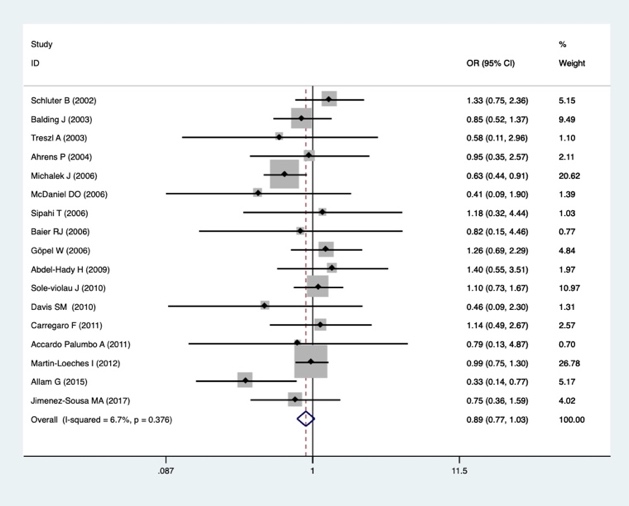


(2) Figure of Egger’s linear regression test


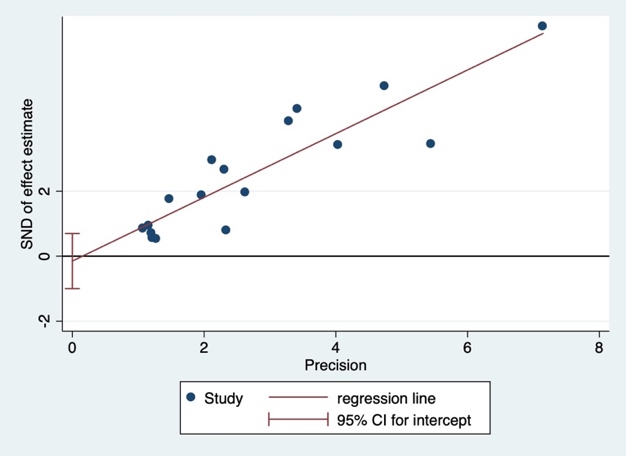


(3) Figure of sensitivity analysis


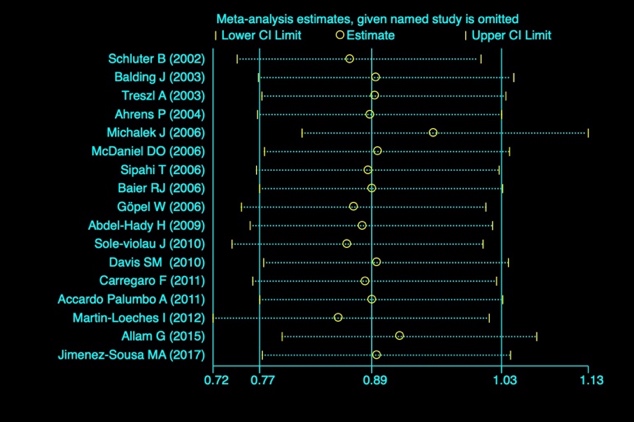


(4) Figure of trial sequential analysis


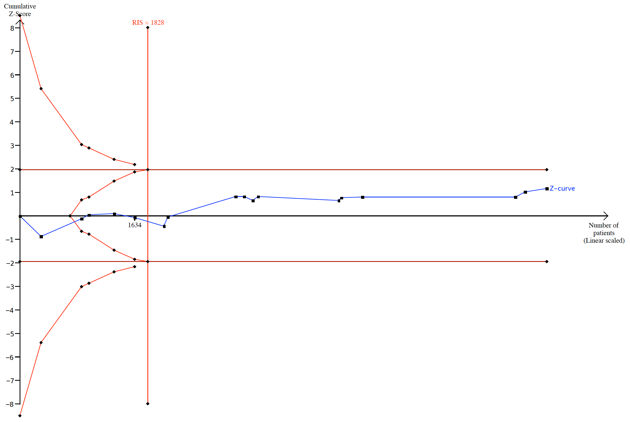


4.3 codominant model (GC vs. GG)

(1) Forest plots


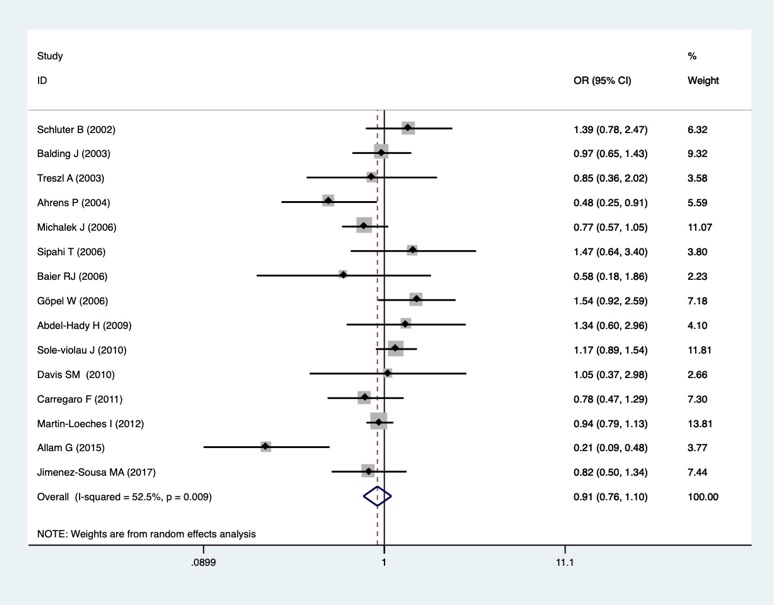


(2) Figure of Egger’s linear regression test


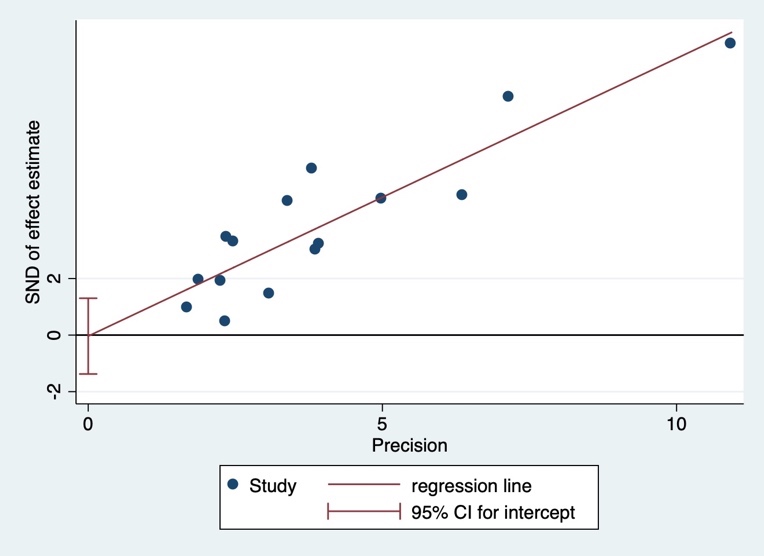


(3) Figure of sensitivity analysis


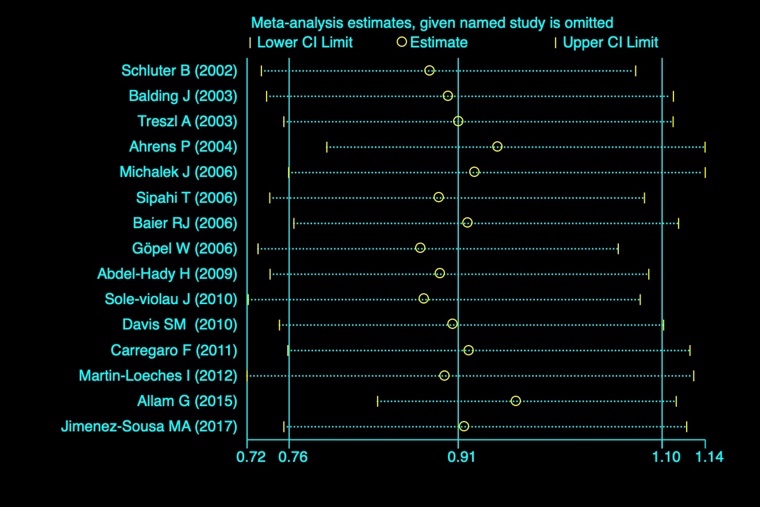


4.4 allelic model

(1) Forest plots


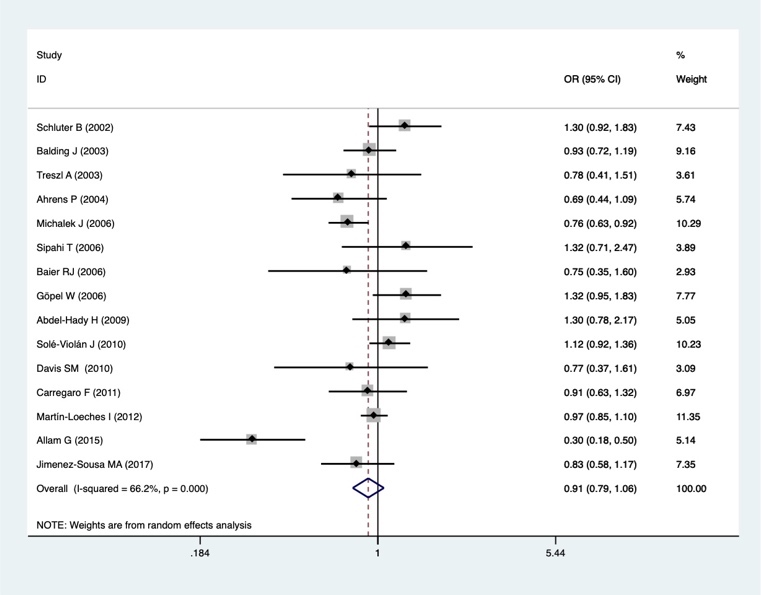


(2) Figure of Egger’s linear regression test


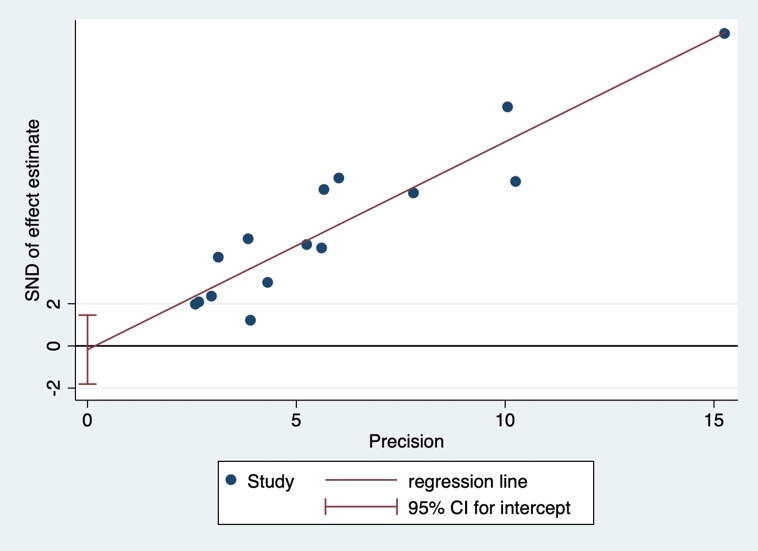


(3) Figure of sensitivity analysis


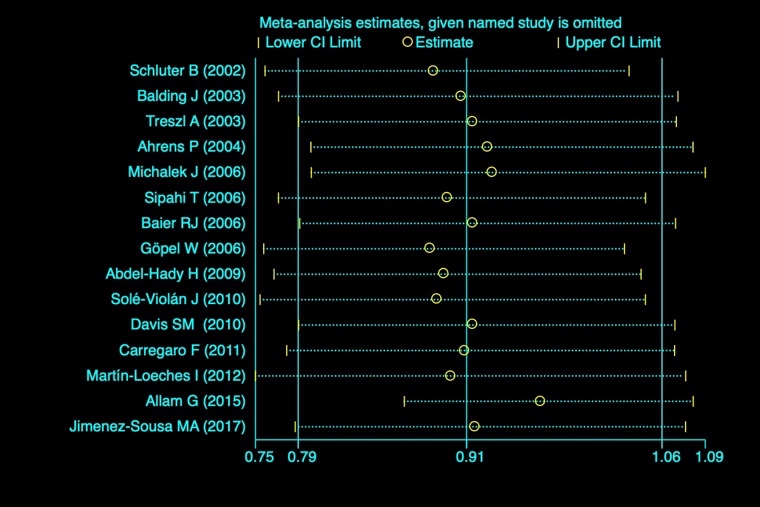


4.5 codominant model (CC vs. GG)

(1) Forest plots


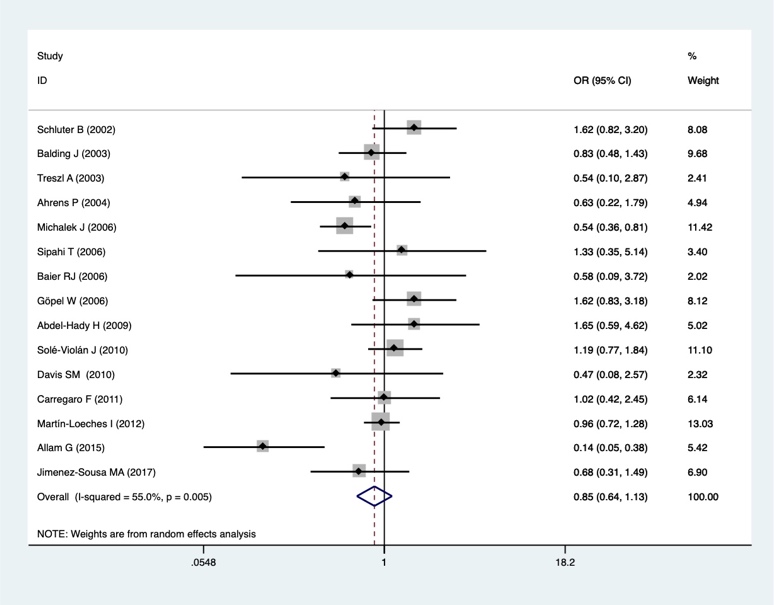


(2) Figure of Egger’s linear regression test


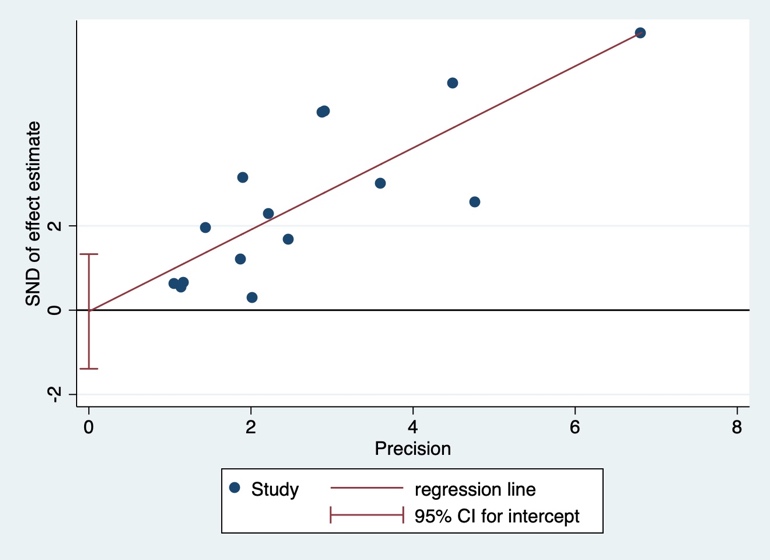


(3) Figure of sensitivity analysis


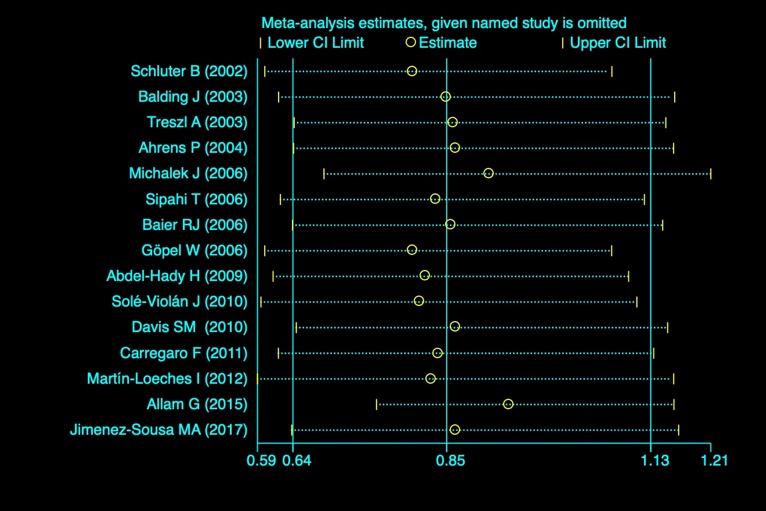


5. Healthy control

5.1 dominant model

(1) Forest plots


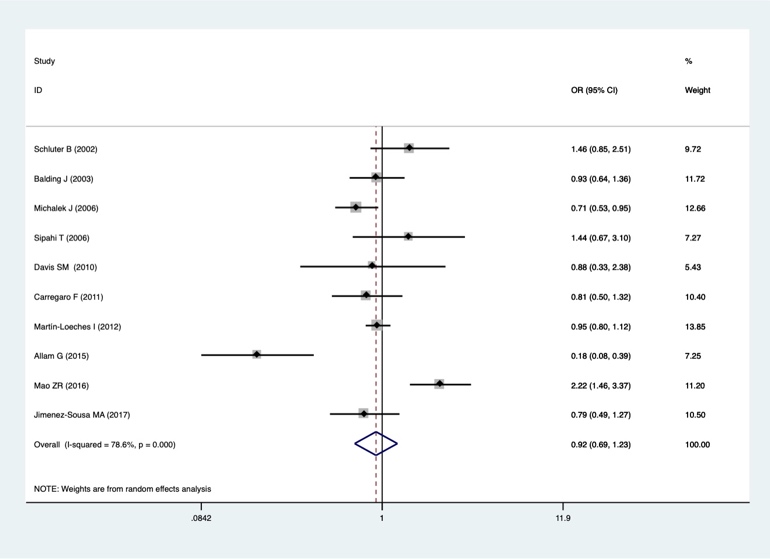


(2) Figure of Egger’s linear regression test


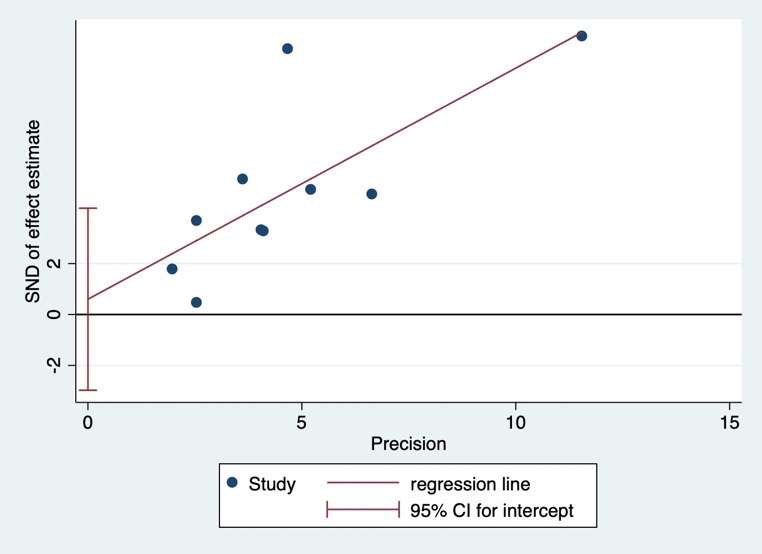


(3) Figure of sensitivity analysis


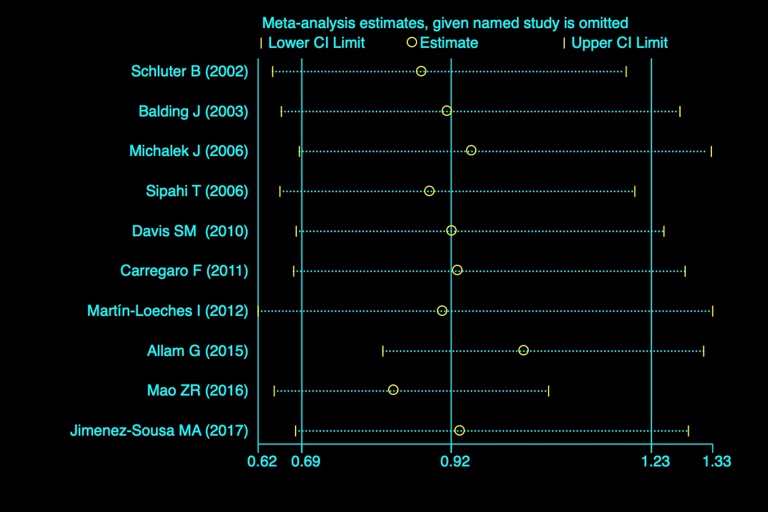


5.2 recessive model

(1) Forest plots


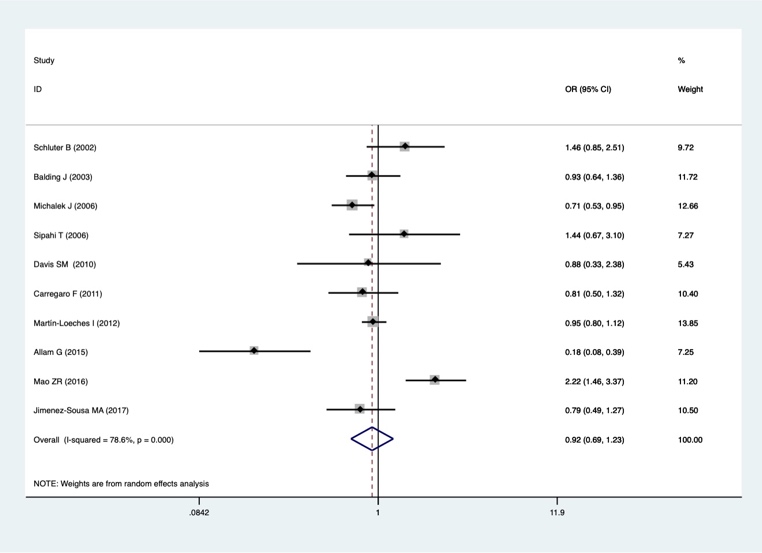


(2) Figure of Egger’s linear regression test


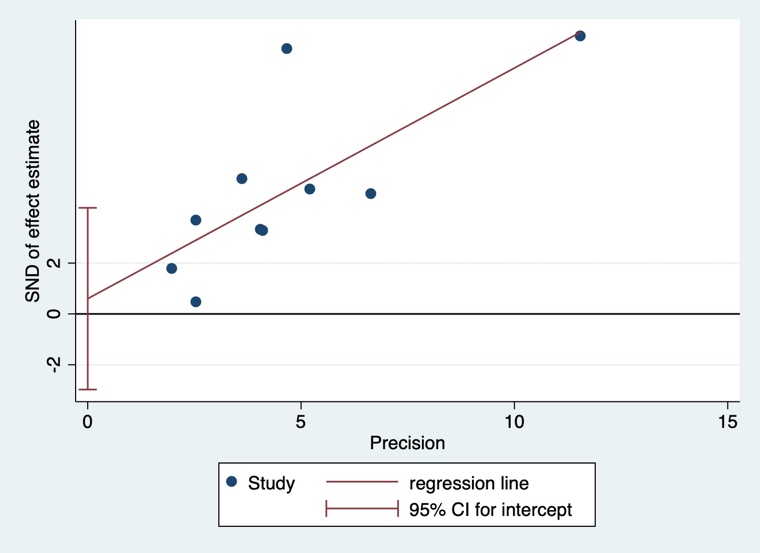


(3) Figure of sensitivity analysis


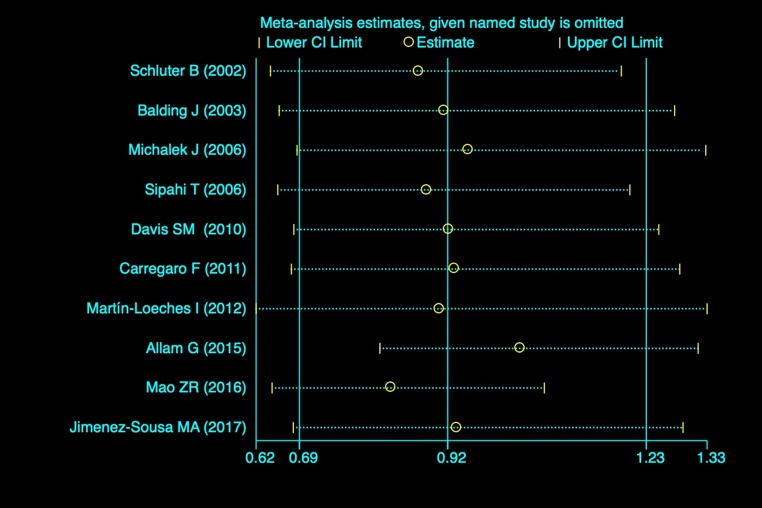


5.3 codominant model(GC vs. GG)

(1) Forest plots


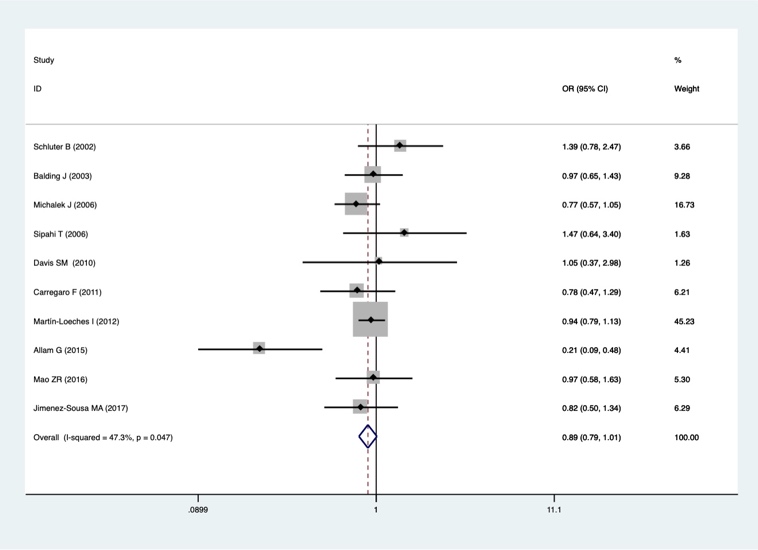


(2) Figure of Egger’s linear regression test


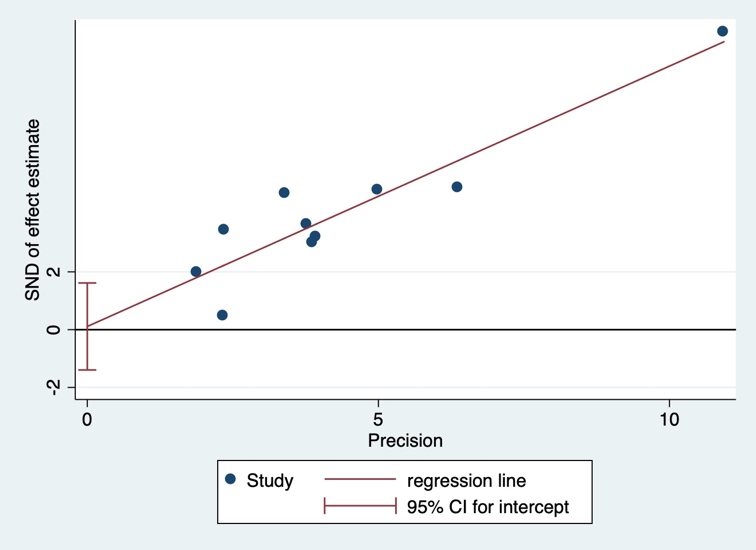


(3) Figure of sensitivity analysis


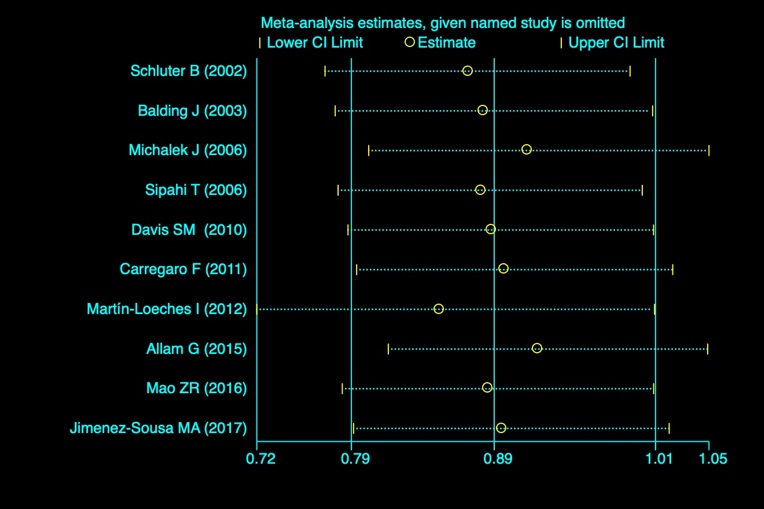


5.4 codominant model(CC vs. GG)

(1) Forest plots


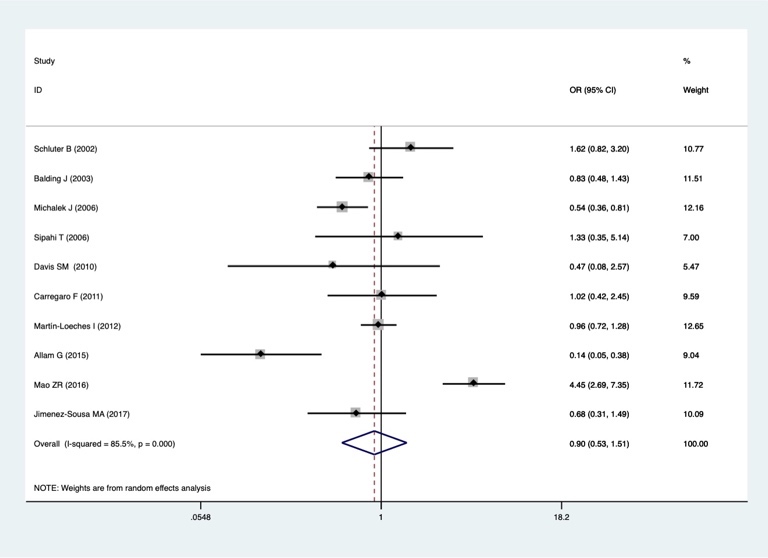


(2) Figure of Egger’s linear regression test


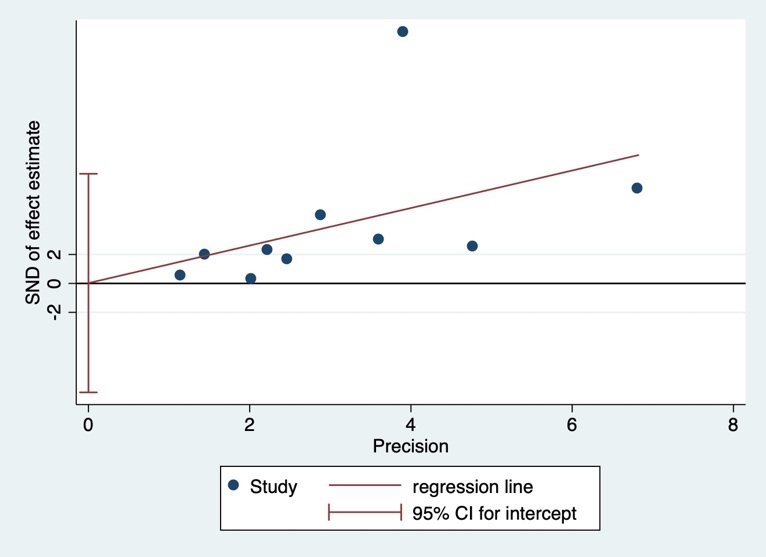


(3) Figure of sensitivity analysis


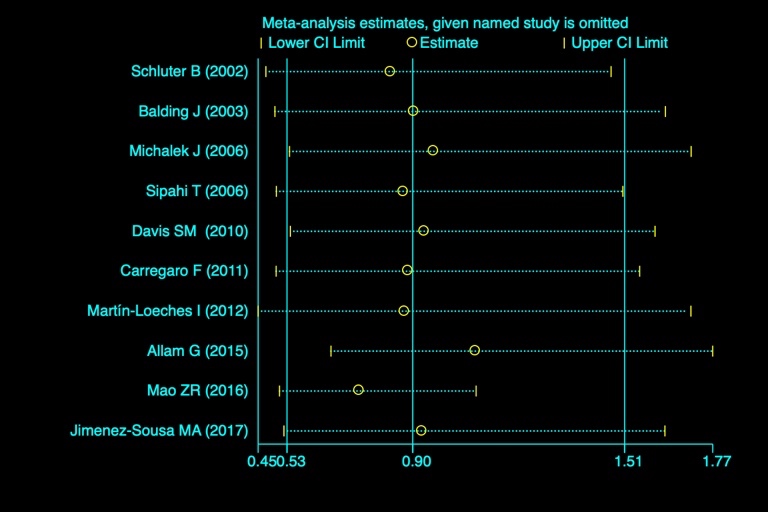


5.5 allelic model

(1) Forest plots


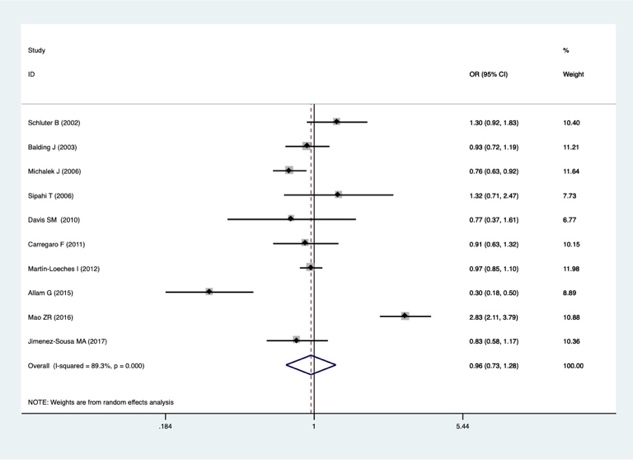


(2) Figure of Egger’s linear regression test


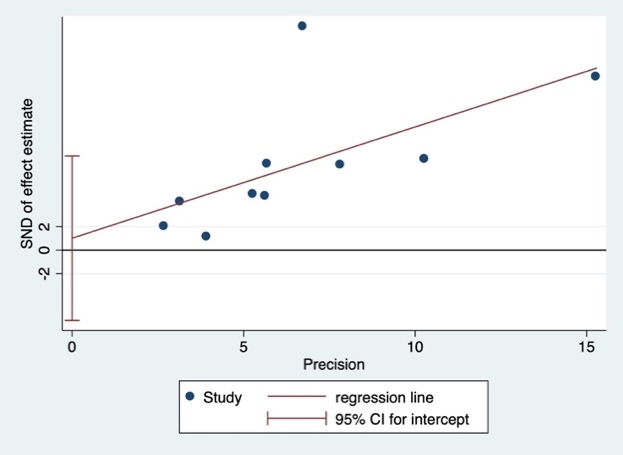


(3) Figure of sensitivity analysis


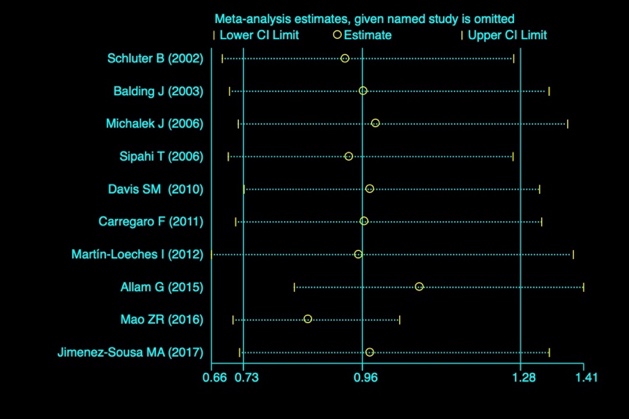


(4) Figure of trial sequential analysis


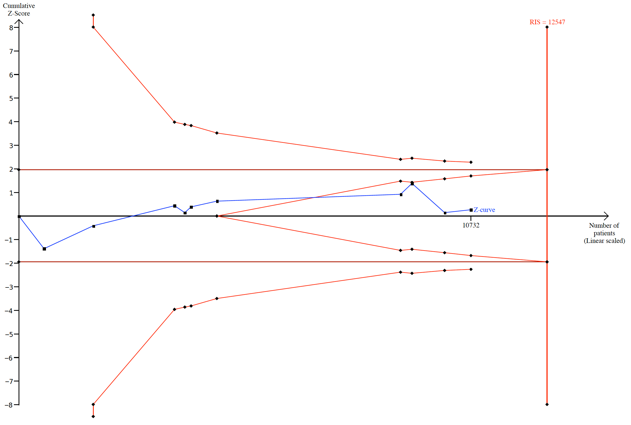


6. Mendelian population

6.1 dominant model

(1) Forest plots


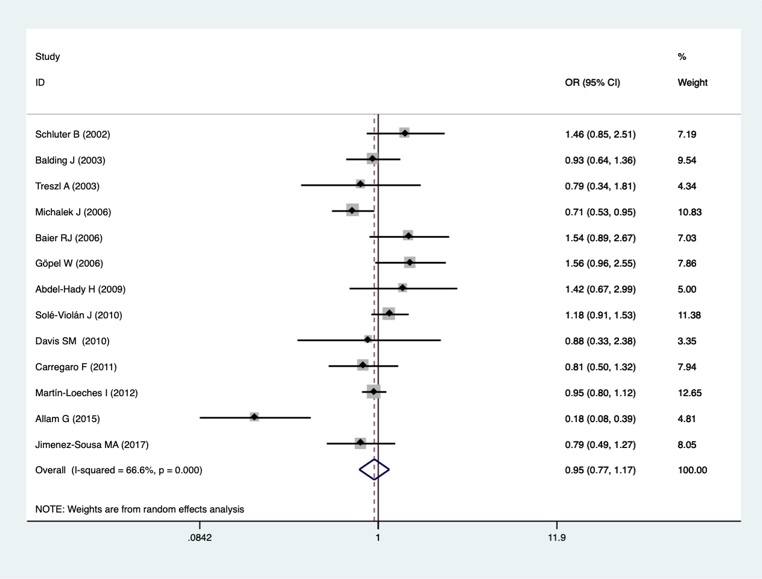


(2) Figure of Egger’s linear regression test


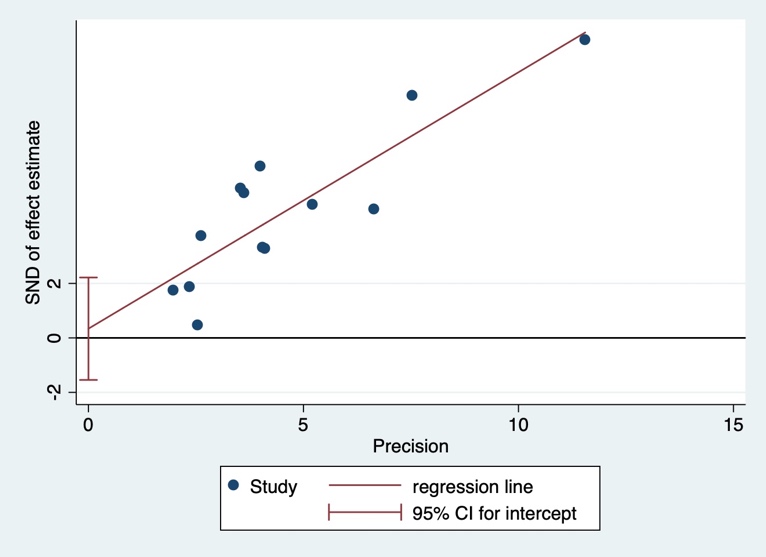


(3) Figure of sensitivity analysis


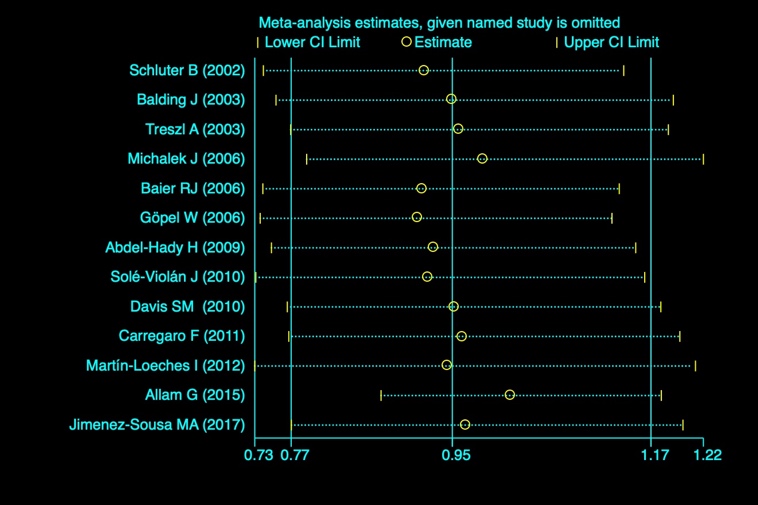


6.2 recessive model

(1) Forest plots


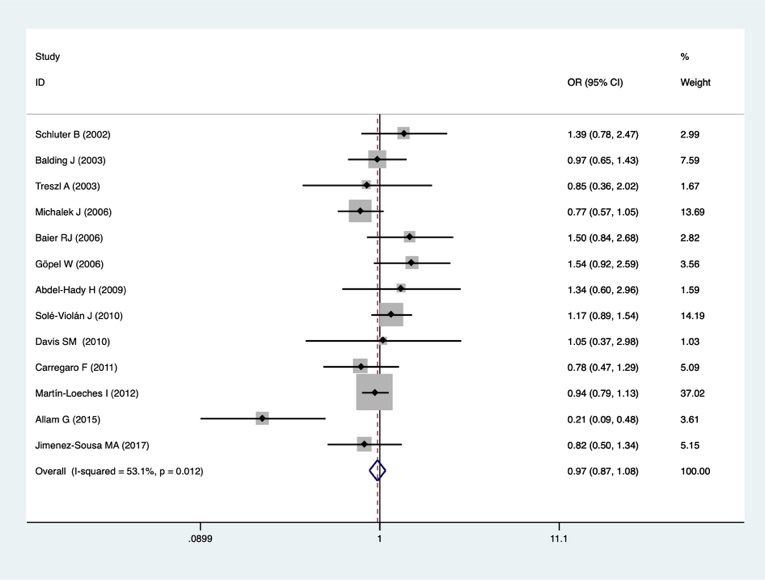


(2) Figure of Egger’s linear regression test


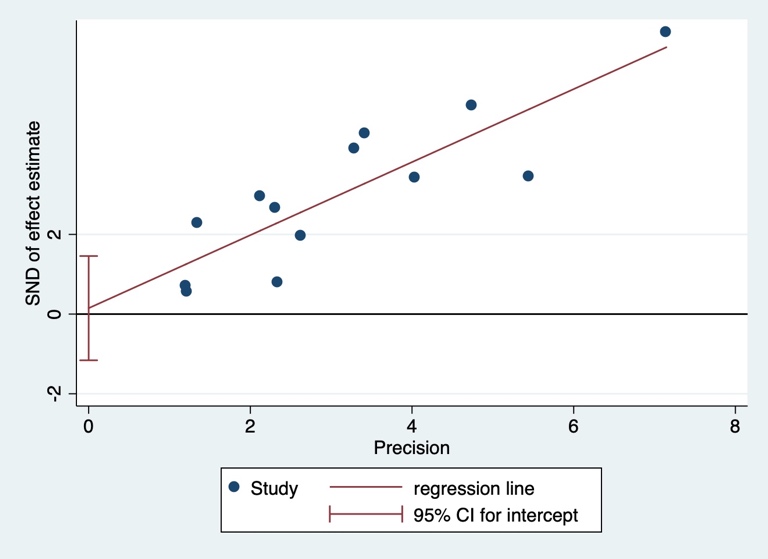


(3) Figure of sensitivity analysis


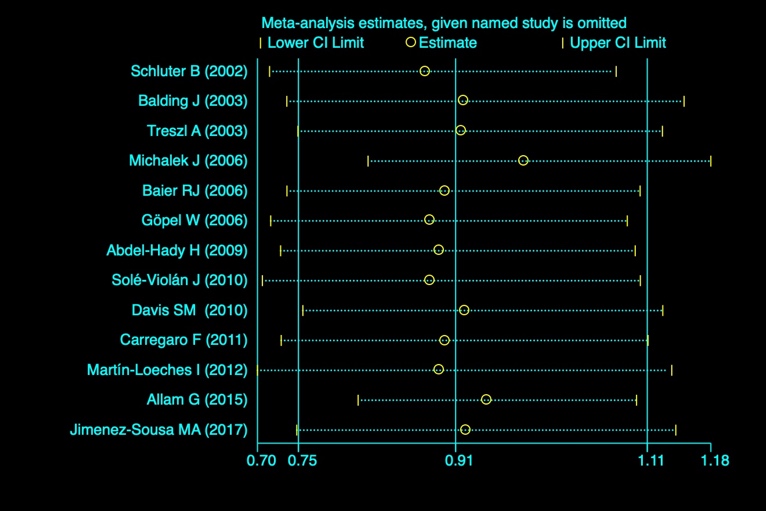


6.3 codominant model (GC vs. GG)

(1) Forest plots


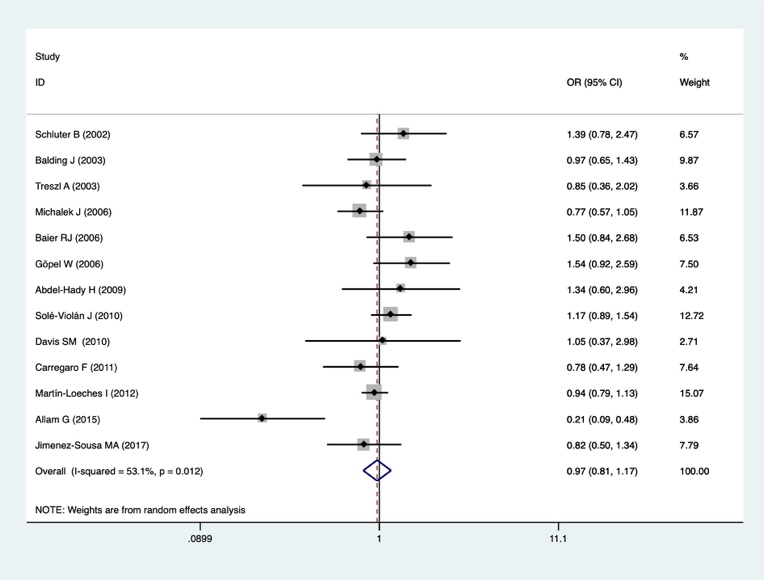


(2) Figure of Egger’s linear regression test


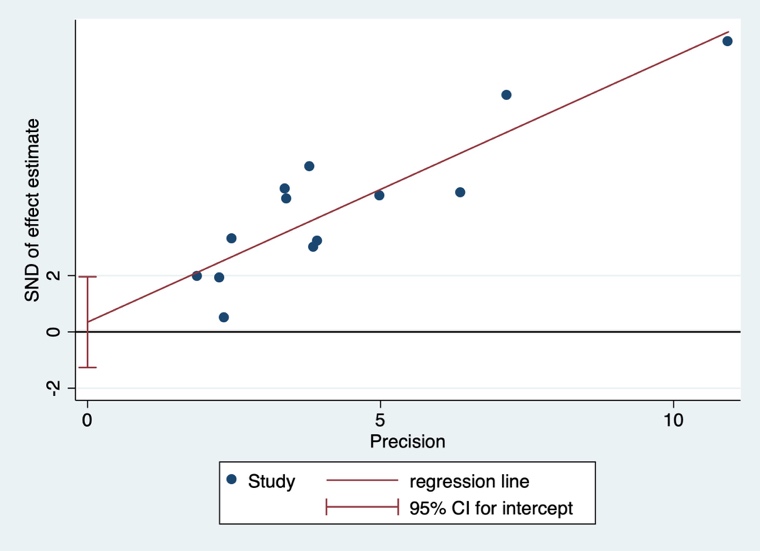


(3) Figure of sensitivity analysis


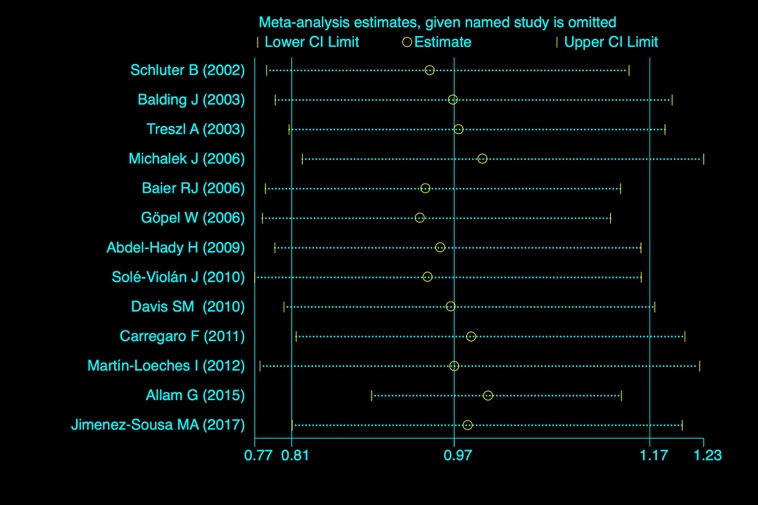


6.4 codominant model (CC vs. GG)

(1) Forest plots


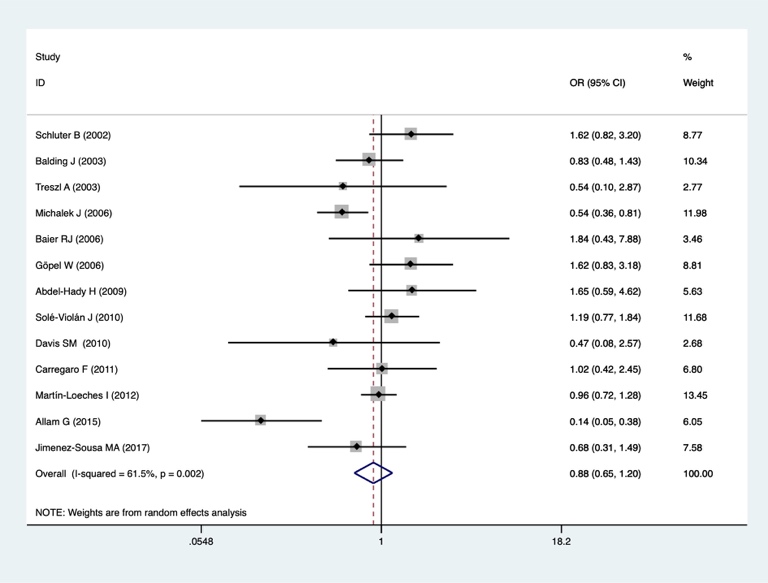


(2) Figure of Egger’s linear regression test


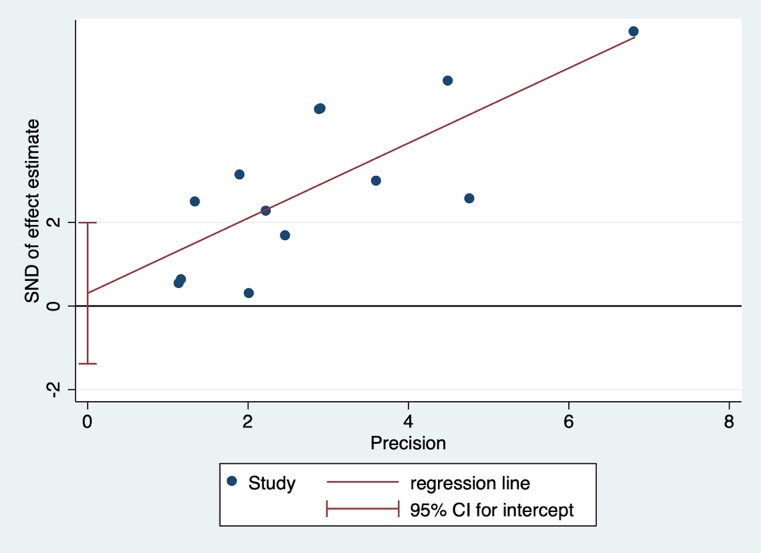


(3) Figure of sensitivity analysis


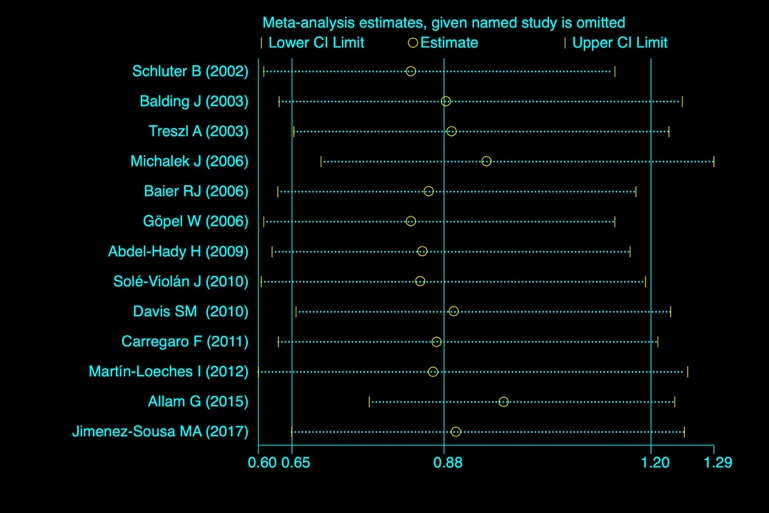


6.5 allelic model

(1) Forest plots


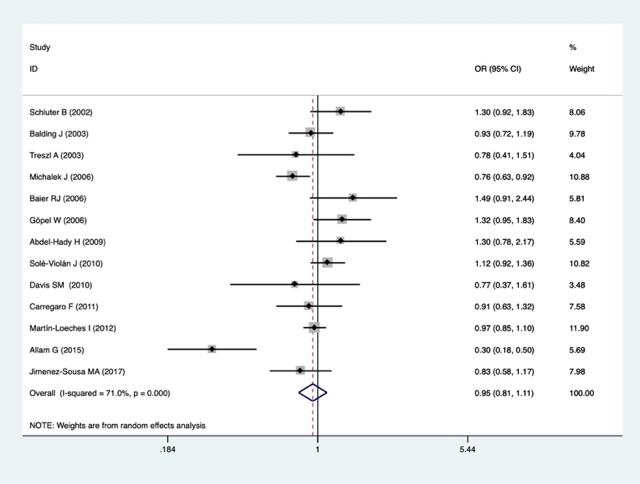


(2) Figure of Egger’s linear regression test


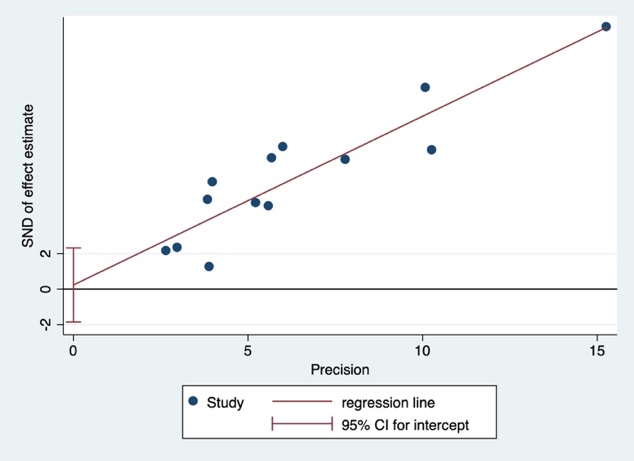


(3) Figure of sensitivity analysis


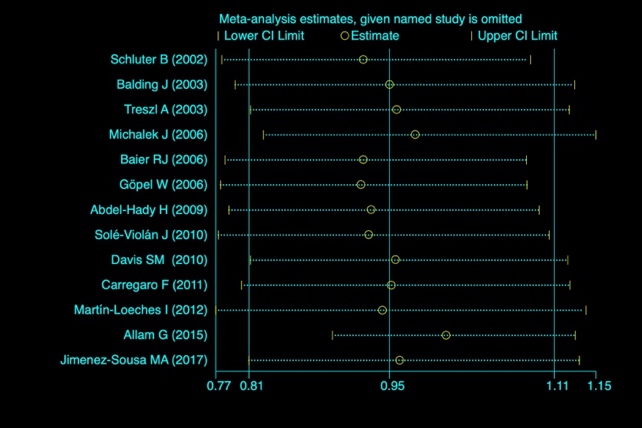


(4) Figure of trial sequential analysis

**
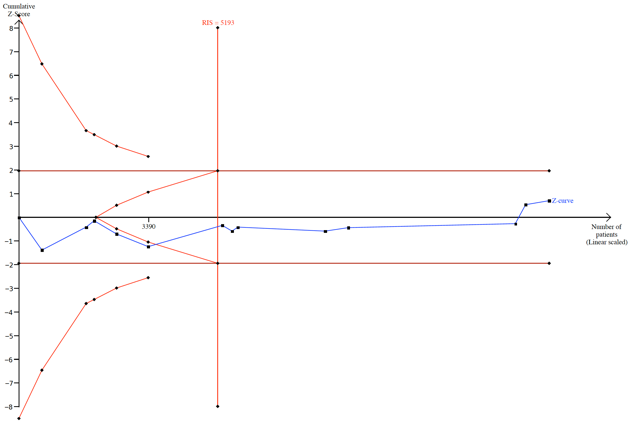
**

**The association between *IL-6*-174 G/C polymorphism and the mortality of sepsis**

1. Overall analysis

1.1 dominant model

(1) Forest plots


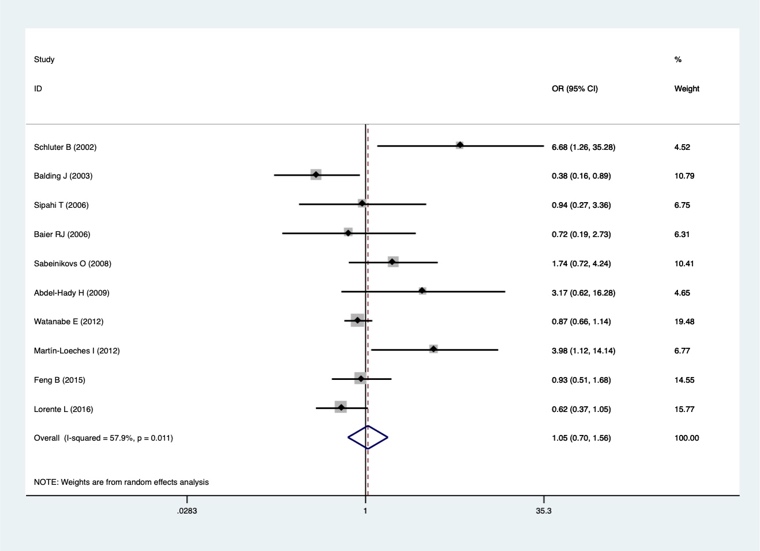


(2) Figure of Egger’s linear regression test


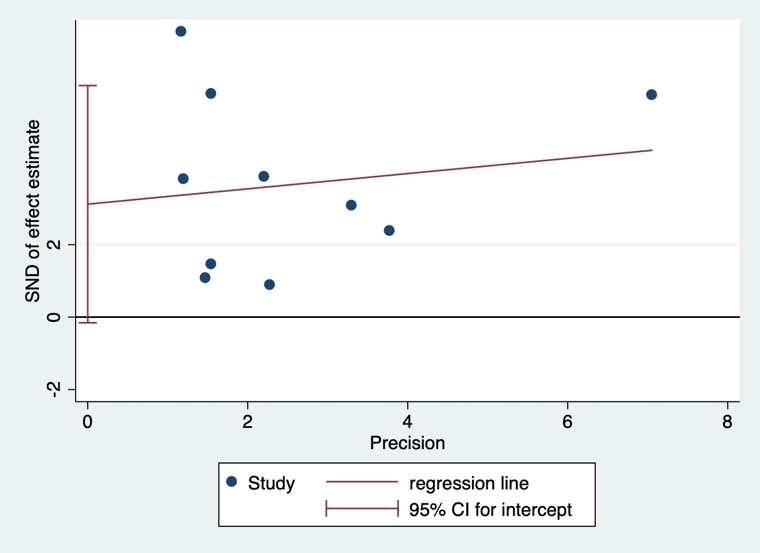


(3) Figure of sensitivity analysis


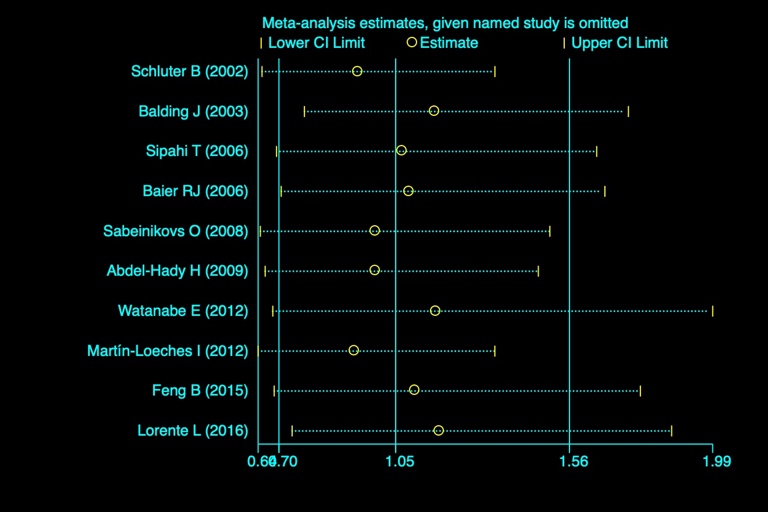


1.2 recessive model

(1) Forest plots


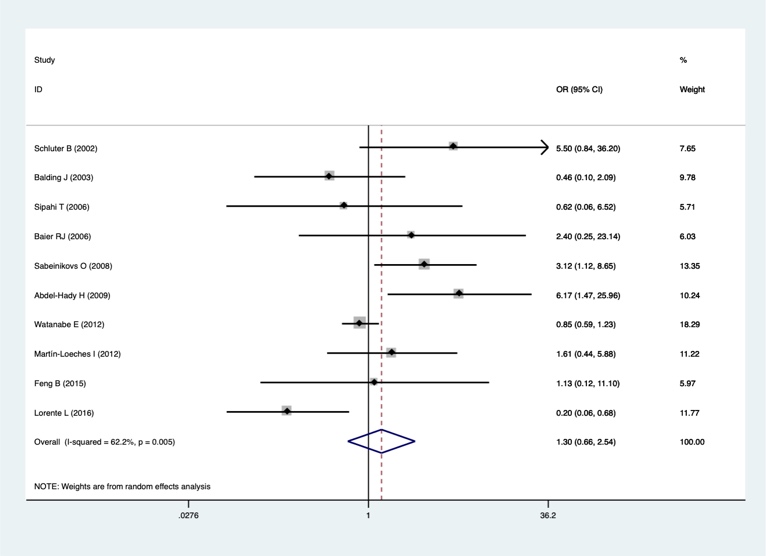


(2) Figure of Egger’s linear regression test


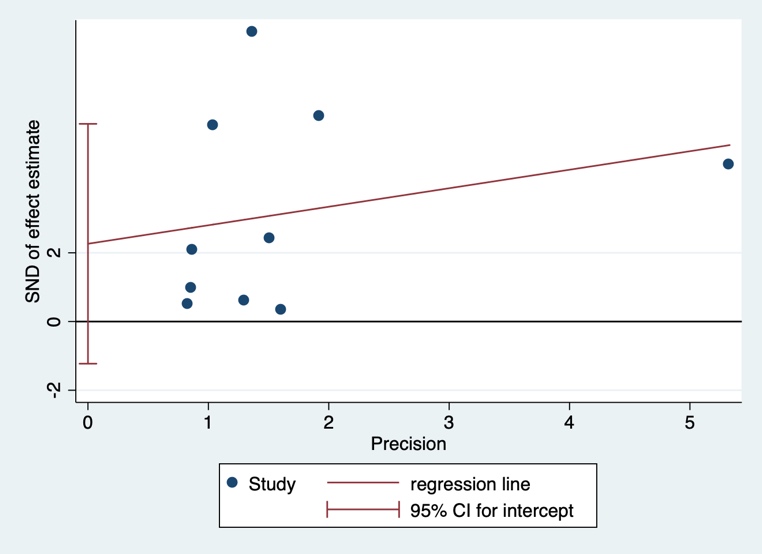


(3) Figure of sensitivity analysis

1.3 codominant model (GC vs. GG)

(1) Forest plots

(2) Figure of Egger’s linear regression test

(3) Figure of sensitivity analysis

1.4 codominant model (CC vs. GG)

(1) Forest plots

(2) Figure of Egger’s linear regression test

(3) Figure of sensitivity analysis

1.5 allelic model

(1) Forest plots

(2) Figure of Egger’s linear regression test

(3) Figure of sensitivity analysis

(4) Figure of trial sequential analysis

2. Non-adult

2.1 dominant model

(1) Forest plots

(2) Figure of Egger’s linear regression test

(3) Figure of sensitivity analysis

2.2 recessive model

(1) Forest plots

(2) Figure of Egger’s linear regression test

(3) Figure of sensitivity analysis

(4) Figure of trial sequential analysis

2.3 codominant model (GC vs. GG)

(1) Forest plots

(2) Figure of Egger’s linear regression test

(3) Figure of sensitivity analysis

2.4 codominant model (CC vs. GG)

(1) Forest plots

(2) Figure of Egger’s linear regression test

(3) Figure of sensitivity analysis

(4) Figure of trial sequential analysis

2.5 allelic model

(1) Forest plots

(2) Figure of Egger’s linear regression test

(3) Figure of sensitivity analysis

(4) Figure of trial sequential analysis

3 Adult

3.1 dominant model

(1) Forest plots

(2) Figure of Egger’s linear regression test

(3) Figure of sensitivity analysis

3.2 recessive model

(1) Forest plots

(2) Figure of Egger’s linear regression test

(3) Figure of sensitivity analysis

3.3 codominant model (GC vs. GG)

(1) Forest plots

(2) Figure of Egger’s linear regression test

(3) Figure of sensitivity analysis

3.4 codominant model (CC vs. GG)

(1) Forest plots

(2) Figure of Egger’s linear regression test

(3) Figure of sensitivity analysis

3.5 allelic model

(1) Forest plots

(2) Figure of Egger’s linear regression test

(3) Figure of sensitivity analysis

(4) Figure of trial sequential analysis

4 Caucasian

4.1 dominant model

(1) Forest plots

(2) Figure of Egger’s linear regression test

(3) Figure of sensitivity analysis

4.2 recessive model

(1) Forest plots

(2) Figure of Egger’s linear regression test

(3) Figure of sensitivity analysis

4.3 codominant model (GC vs. GG)

(1) Forest plots

(2) Figure of Egger’s linear regression test

(3) Figure of sensitivity analysis

4.4 codominant model (CC vs. GG)

(1) Forest plots

(2) Figure of Egger’s linear regression test

(3) Figure of sensitivity analysis

4.5 allelic model

(1) Forest plots

(2) Figure of Egger’s linear regression test

(3) Figure of sensitivity analysis

(4) Figure of trial sequential analysis
